# The rise and evolution of cancer mechanobiology: a bibliometric trajectory of three decades of research

**DOI:** 10.3389/fphar.2026.1699709

**Published:** 2026-03-16

**Authors:** Boyan Liu, Xufeng Liu, Yue Wang, Xiao Han, Xiyu Dang

**Affiliations:** 1 Hospital of Chengdu University of Traditional Chinese Medicine, Chengdu, China; 2 Ninth Hospital of Xi’an, Xi’an, China; 3 Shanxi Academy of Traditional Chinese Medicine, Xi’an, China

**Keywords:** bibliometric analysis (BA), cancer immunotherapy, cancer mechanobiology, extracellular matrix, knowledge map, tumor microenvironment

## Abstract

**Background:**

A growing body of research indicates that mechanobiology plays a pivotal role in cancer pathogenesis and holds considerable therapeutic potential. However, a comprehensive bibliometric analysis of this interdisciplinary field is lacking, partly due to challenges in cross-database data integration. In this study, we aim to construct a systematic knowledge map of cancer mechanobiology to delineate its research progress, core structure, and emerging trends.

**Methods:**

In this study, we integrated 1,947 publications from the Web of Science (WoS) Core Collection and Scopus (1976–2025). To address cross-database heterogeneity, we developed a novel, customized, multi-stage data-standardization workflow combining a bespoke Python parsing engine with fuzzy string matching algorithms and manual verification. The unified dataset was analyzed using CiteSpace, VOSviewer, and Bibliometrix.

**Results:**

The United States and China are the most prolific countries, while the University of California system is the most productive institution. Valerie M. Weaver is the most published author, while Matthew J. Paszek is the most co-cited, indicating foundational influence. Cell is the most influential journal based on co-citation frequency. Keyword analysis reveals a thematic evolution from “extracellular matrix stiffness” and “mechanotransduction” to frontier areas such as “cancer immunotherapy” and “YAP signaling protein.”

**Conclusion:**

In this study, we construct a comprehensive bibliometric map of cancer mechanobiology. Our findings elucidate the developmental trajectory and research hotspots of the field, providing a data-driven reference for future investigations, international collaborations, and clinical translation of physical oncology.

## 1 Introduction

Mechanobiology, the field dedicated to studying how cells generate, sense, and respond to mechanical forces, plays a pivotal role in understanding how cells and tissues form, develop, and remodel during development and disease (Nelson et al., 2024). Since the pioneering work of Y.C. Fung in biomechanics during the 1960s, mechanobiology has expanded its theoretical framework from the macroscopic tissue level to the microscopic molecular level, fostering a deep understanding of the ‘stress–growth’ relationship (Fung, 2013). Technological advancements have further broadened the scope of mechanobiology, extending it from traditional biomechanics to cellular and molecular investigations and establishing it as a critical lens for understanding life processes (Jiang, 2023). Within cancer research, mechanobiology offers a unique perspective, elucidating the mechanisms by which mechanical factors within the tumor microenvironment (TME) influence tumorigenesis, progression, metastasis, and therapeutic response (Xin et al., 2023; Zhang Y. et al., 2025).

As a global public health challenge, the study of cancer has evolved from early morphological descriptions to a complex interdisciplinary systems-level pursuit. Its growing integration with fields such as immunology, in particular, has shown immense potential (Graciotti and Kandalaft, 2025; Roerden and Spranger, 2025). Traditional cancer research, although it has made significant strides, has predominantly focused on molecular mechanisms at the genomic, proteomic, and metabolomic levels. Nevertheless, cancer research continues to face challenges, including high tumor heterogeneity, drug resistance, and a lack of therapeutic specificity (Bernards et al., 2020). In recent years, research on tumor biomechanics has gained traction, revealing the impact of key mechanisms—such as alterations in cancer cell stiffness, regulation by extracellular matrix (ECM) rigidity, and mechanical signal transduction—on tumor progression (Urcun et al., 2022). For example, the stiffness of the tumor ECM can be 5–10 times greater than that of normal tissue, promoting the acquisition of cancer stem cell properties, epithelial–mesenchymal transition (EMT), and metastasis via the integrin–Yes-associated protein/transcriptional coactivator with PDZ-binding motif (YAP/TAZ) pathway (Zhang and Zhang, 2025). Concurrently, cancer cells can evade immune detection by reducing their own stiffness, which disrupts the formation of the immune synapse (Park et al., 2025). These findings have inspired several therapeutic hypotheses, including the use of matrix-associated enzymes to promote ECM normalization, controlling tumor progression by reducing hyaluronic acid (HA) expression in the ECM, and enhancing T-cell immunotherapy by softening the ECM (Karalis et al., 2019; Lei et al., 2021; Zhu et al., 2023). Such discoveries indicate that mechanical factors play an indispensable role in cancer development, and mechanobiology, as a growing interdisciplinary field, provides a new theoretical framework and novel research tools for cancer investigation.

Critically, the evolution of cancer mechanobiology has not been linear but has proceeded through three conceptually distinct yet interconnected phases, each representing a pivotal turning point that has fundamentally reshaped the field’s questions and methods. The first phase, spanning approximately from the 1970s through the late 1990s, was characterized by the study of macroscopic mechanical phenomena: researchers documented that solid tumors exhibit elevated interstitial fluid pressure, increased tissue stiffness, and abnormal solid stress, establishing that the physical tumor microenvironment is profoundly different from that of normal tissue (Jain, 1987; Helmlinger et al., 1997; Paszek et al., 2005). The second phase was triggered by molecular dissection of these macroscopic observations. Landmark studies by Paszek et al. (2005) and Levental et al. (2009) demonstrated that extracellular matrix stiffness directly drives malignant transformation via integrin-mediated signaling (Paszek et al., 2005; Levental et al., 2009), while the discovery by Dupont et al. (2011) that YAP/TAZ serve as universal mechanotransducers provided a molecular node linking physical cues to transcriptional programs governing proliferation, stemness, and drug resistance (Dupont et al., 2011). This mechanomolecular paradigm elevated the field from phenomenological description to mechanistic explanation. The third and currently emerging phase is defined by the imperative of clinical translation: mechanobiological insights are being integrated into immunotherapeutic strategies as it is now recognized that ECM stiffness impairs T-cell infiltration and immune synapse formation and that mechanically normalizing the tumor stroma can enhance the efficacy of checkpoint blockade immunotherapy (Mai et al., 2024). This three-phase trajectory—from macroscopic phenomena, through YAP/TAZ-centered micromolecular mechanisms, to immune-mechanobiology-based clinical intervention—constitutes the essential historical and conceptual context that motivates the present study. Understanding where the field stands within this trajectory, which turning points have generated the most enduring intellectual influence, and which frontier directions are accruing the fastest momentum are precisely the kind of questions that a systematic bibliometric analysis is uniquely positioned to answer.

Despite a surge in related studies, this field lacks a comprehensive and quantitative assessment of its evolution, knowledge structure, and emerging trends. Although narrative reviews periodically summarize specific aspects of cancer mechanobiology, the absence of a systematic bibliometric analysis prevents researchers from gaining a holistic understanding of the field’s trajectory, identifying knowledge gaps, and recognizing the collaborative networks that drive innovation. Such an analysis is particularly crucial as the field matures and seeks to translate mechanobiological insights into therapeutic strategies.

Bibliometric analysis is a research methodology that utilizes quantitative methods to reveal the knowledge structure, research trends, and developmental patterns of scientific disciplines (Ninkov et al., 2022). Recently, bibliometric analysis has experienced significant methodological advancements and widespread application across diverse medical fields. The integration of machine-learning algorithms and advanced informatics has propelled this methodology beyond simple citation counting, enabling unsupervised clustering and global-scale evaluations of complex biomedical landscapes. For instance, recent machine-learning-driven bibliometric studies have successfully been utilized to map the global concerns and future directions of large language models in medicine (Guo et al., 2025a) and systematically profile the decades-long scientific landscape of immune-related adverse events in neoadjuvant immunotherapy for patients with perioperative cancer (Guo et al., 2025b). Furthermore, these advanced analytical frameworks have recently been deployed to track the expanding application of deep learning architectures in cancer diagnosis and precision oncology (Wang R. et al., 2024). These modern applications highlight the robust capability of bibliometrics to distill massive datasets into actionable clinical insights, identify emerging research hotspots, and guide therapeutic strategies. In this study, we utilize a bibliometric approach, combining visualization tools such as CiteSpace and VOSviewer, to conduct a systematic analysis of the literature at the intersection of mechanobiology and cancer. The objective is to map the research hotspots, developmental trends, collaborative networks, and intellectual foundations of this domain (Thompson and Walker, 2015). By analyzing metrics such as the publication volume, citation frequency, co-authorship networks, and keyword co-occurrence, we aim to comprehensively assess the contributions, limitations, and future directions of mechanobiology in cancer research, thereby providing a scientific basis for further investigation and clinical translation.

Although previous reviews have extensively summarized the biological mechanisms of cancer mechanobiology, such as the role of matrix stiffness in regulating YAP/TAZ signaling and its impact on tumor progression (Gajda et al., 2025), and explored the potential of biomechanical signals in drug responsiveness and immunotherapy, a systematic and quantitative bibliometric analysis from a pharmacological perspective remains lacking. Recent advances have highlighted the concept of “mechanotherapeutics”—agents that target mechanotransduction pathways or modulate cellular stiffness to enhance treatment efficacy (Wu et al., 2025). For instance, drugs such as losartan, pirfenidone, and tranilast have been shown to remodel the tumor microenvironment by reducing solid stress and improving vascular perfusion, thereby potentiating chemotherapy and immunotherapy (Wu et al., 2025). Moreover, emerging evidence indicates that the pharmacological manipulation of mechanosensitive ion channels and cytoskeletal regulators may offer novel therapeutic avenues for refractory tumors (Peng et al., 2025). Despite these promising developments, with a specific focus on their therapeutic implications, it remains unclear how mechanical signals are being targeted, what drug classes are involved, and which molecular mechanisms are being exploited for clinical translation. Therefore, we aim to fill this gap by providing the first bibliometric analysis that integrates the pharmacological dimension into the broader framework of cancer mechanobiology. By identifying the key drugs, mechanosensitive targets, and emerging therapeutic strategies, we not only delineate the evolution of this interdisciplinary field but also offer a data-driven roadmap for future drug development and precision mechanomedicine.

The interdisciplinary field of oncology and biomechanics is rapidly advancing, yet a systematic, quantitative analysis of its knowledge structure and developmental trajectory is currently lacking. Given that cancer mechanobiology resides at the intersection of oncology, biomechanics, materials science, and immunology, the pertinent literature is highly fragmented across disparate academic databases. Relying on a single database inherently introduces coverage bias; for example, the Web of Science (WoS) Core Collection tends to favor high-impact basic research, whereas Scopus provides broader coverage of applied engineering and biomedical innovations. Consequently, the integration of data from multiple platforms is not merely an option but a fundamental prerequisite for a holistic analysis. However, conventional bibliometric studies and existing built-in software converters frequently fail to manage the significant data heterogeneity between WoS and Scopus. Direct data merging often results in misaligned cited references (CRs), fragmented author affiliations (C1), and inflated collaboration metrics. As a result, current bibliometric research conventionally adopts the approach of analyzing different databases separately to cross-validate the findings. To overcome the severe limitations of the existing narrative reviews and traditional bibliometric software, this study elevates data fusion to a core methodological driver. We engineered a sophisticated, customized, multi-stage Python standardization pipeline to harmonize these cross-database discrepancies. This tailored computational approach is imperative for substantially mitigating structural data biases, thereby enabling the construction of a high-fidelity, genuinely comprehensive knowledge graph of the physical tumor microenvironment. This study focuses on identifying the core research communities, leading institutions, and international collaboration networks, which are of significant importance for fostering academic exchange and optimizing the allocation of research resources.

## 2 Materials and methods

### 2.1 Database and search strategy

The WoS Core Collection and Scopus are the two most widely used and authoritative databases in bibliometric research, providing comprehensive and structured metadata essential for scientometric network analysis (Pranckute, 2021). Recent analyses indicate a significant but incomplete overlap between the Scopus and WoS Core Collection databases (Clarivate Analytics’s Web of Science Core Collection); 53% of publications in Scopus were indexed by WoS, while 74% of WoS publications were indexed by Scopus (Abdulhayoglu and Thijs, 2018). However, studies have demonstrated that discrepancies in their journal coverage can lead to divergent outcomes in bibliometric analyses (Harzing and Alakangas, 2016; Mongeon and Paul-Hus, 2016). Furthermore, the two databases are considered complementary, each offering unique strengths in the linguistic and geographical scope (Sánchez et al., 2017; Vera-Baceta et al., 2019). Therefore, to construct a comprehensive and unbiased bibliographic dataset, we implemented a systematic search and screening protocol that leverages the distinct advantages of both WoS and Scopus.

#### 2.1.1 Data collection and screening

Some bibliometric studies suffer from incomplete data acquisition due to institutional subscription limitations. To mitigate this, our study utilized multiple access channels to ensure the broadest possible search coverage. A systematic search was conducted on 10 August 2025, covering all publications from 1 January 1976 up to the search date. The search strategy, optimized for the syntax of each database, was designed to capture the core terminology, such as “mechanobiology” and “cancer,” from titles, abstracts, and keywords. The WoS query was as follows: TS=(mechanobiology OR “mechanical stress” OR “matrix stiffness” OR “mechanotransduction” OR “cell mechanics” OR “physical oncology”) AND TS=(cancer OR tumor OR neoplasm OR carcinoma). The corresponding Scopus query was as follows: TITLE-ABS-KEY (mechanobiology OR “mechanical stress” OR “matrix stiffness” OR “mechanotransduction” OR “cell mechanics” OR “physical oncology”) AND TITLE-ABS-KEY (cancer OR tumor OR neoplasm OR carcinoma).

**FIGURE 1:**
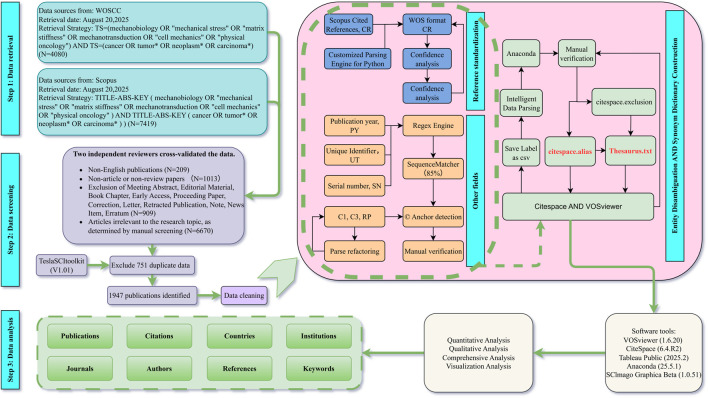
Flowchart of the study.

The initial search yielded 4,080 records from WoS and 7,419 from Scopus. To minimize selection bias, these records subsequently underwent a rigorous two-stage screening process conducted in parallel by two independent reviewers. The first stage involved filtering by document type, retaining only journal articles and reviews. Subsequently, the reviewers manually assessed the titles and abstracts of the remaining documents for their direct relevance to the scope of this study. Any disagreements were resolved through consensus discussion. This refined screening process led to a curated dataset of 1,080 WoS and 1,618 Scopus documents for subsequent processing. Figure 1 shows the flowchart.

### 2.2 Data cleaning and processing

In most bibliometric studies, data are acquired without subsequent meticulous cleaning. Specifically, comma-separated values (CSVs) files exported from the Scopus database and text (TXT) files from the WoS Core Collection are difficult to reconcile, and relying solely on the Scopus-to-WoS conversion function within CiteSpace is insufficient to ensure data quality. Therefore, following the initial acquisition and screening, we executed a comprehensive, multi-stage cleaning and standardization protocol on the refined dataset. This protocol was designed to ensure the highest data quality and prepare the corpus for robust scientometric analysis, encompassing manual validation, cross-database deduplication, reference standardization, and entity disambiguation.

#### 2.2.1 Manual validation and cross-database deduplication

To establish a baseline of data reliability, we implemented a stringent validation protocol for the data extraction process. Two independent reviewers extracted and verified key bibliographic information—including the journal, title, author, institution, abstract, and keywords—according to predefined criteria to mitigate selection bias. The two resulting datasets were then systematically cross-validated to identify and rectify any inconsistencies, with all discrepancies resolved by consulting the original publications. This process ensured a transparent and reproducible audit trail for the refined data.

To identify and remove duplicate records across databases while concurrently supplementing incomplete entries, we processed the refined dataset using the open-source toolkit TeslaSCI (Nikolić et al., 2024). This tool utilizes a user-configurable, similarity-based matching methodology, leveraging a standardized Damerau–Levenshtein distance algorithm to compute similarity scores for key bibliographic fields such as title, author, digital object identifier (DOI), and publication year. Based on preset similarity thresholds, the toolkit partitions records into three distinct sets—recommended matches, potential matches, and non-matches—facilitating a semi-automated deduplication and data supplementation workflow. To construct the definitive Scopus dataset, we extracted and integrated all records identified as originating from Scopus across these categories. Data harmonization procedures were applied during integration to unify column structures and ensure data integrity, yielding a final set of 867 comprehensively supplemented and unique Scopus records. Correspondingly, we constructed the authoritative WoS dataset by compiling a list of 1,080 unique WoS identifiers (UTs) from the toolkit’s output and using this list to precisely filter the full records downloaded from the WoS platform. This process included internal deduplication to ensure the uniqueness of the final dataset, requiring approximately 30 min for manual verification and matching. Subsequent preliminary validation in CiteSpace and VOSviewer confirmed that both datasets were fully parsable by each software application.

#### 2.2.2 Standardization of cited references

To ensure the rigor and reproducibility of our scientometric analysis, we developed and implemented a custom, multi-stage, semi-automated process to handle and standardize the CRs exported from the Scopus database. The raw Scopus CR field is characterized by widespread format heterogeneity, missing critical information, and parsing errors—issues that can severely compromise the validity of subsequent co-citation network analysis and citation metrics. Our designed process systematically addresses these challenges to ensure high data integrity.

This procedure was divided into two main stages. The first stage included automated parsing and confidence scoring. At the core of this stage was a bespoke, Python-based parsing engine tasked with the initial conversion of 61,948 raw CR strings into the standard WoS format. The engine utilized a hierarchical cascade of regular expressions to robustly extract key metadata—including first author, publication year, journal, and DOI—from each citation. For example, DOI extraction utilized a three-tier priority matching strategy (e.g., targeting standard prefixes ‘r (?:doi:|DOI:)?\s*(10\.\d{4,}/[^\s,;)]+)’ before attempting bare formats). Similarly, author names and publication years (PYs) were standardized using progressive regex patterns to accommodate missing or non-standard characters, ensuring seamless conversion into the WoS CR formats. A critical feature of the engine was its sophisticated, multi-strategy journal name normalization module, which operated on three logical tiers: i. direct mapping against a predefined dictionary of several hundred core journals; ii. fuzzy string matching using the difflib.SequenceMatcher algorithm (similarity threshold >0.85); and iii. programmatic rule-based generation of compliant journal abbreviations when the preceding methods failed. Notably, the code performed a quantitative quality assessment for each parse, dynamically assigning a confidence score based on a structural integrity weighting system. A flawless WoS-formatted reference received a base score of 100. Strict penalties were assigned that were essential for CiteSpace co-citation analysis: missing year (−15), missing first author (−20), and missing journal name (−25). Therefore, a score of ≥85 rigorously ensures that all three indispensable nodes for co-citation clustering are successfully extracted, requiring zero manual intervention. This automated step demonstrated high efficacy, successfully processing 61,152 references (98.7%) that were classified as high-confidence (score ≥85) results.

The second stage involved manual review and data correction, which is a process that required a total of 11 h. The remaining 796 low-to-medium confidence and failed records from the first stage were systematically exported to a dedicated Excel workbook, initiating a semi-automated human-in-the-loop validation workflow. In this stage, a researcher verified the records against authoritative databases, such as Google Scholar and PubMed. By design, the workflow enhanced efficiency and accuracy, as the reviewer was not required to manually edit disparate fields but could instead paste the correct, complete reference string into a designated ‘complete reference paste’ column and mark the ‘review status’ as “COMPLETED” or “REJECTED.”

Finally, the high-confidence results from stage one were integrated with the manually curated data from stage two. The script prioritized and re-parsed references marked as “COMPLETED” to ensure that these “gold-standard” data were precisely converted into the unified WoS format. Subsequently, the script reintegrated all high-confidence and manually corrected standardized references back into their corresponding source paper records, completely replacing the original CR field with a clean semicolon-delimited string. This rigorous process yielded a high-quality dataset with a 99.4% reference conversion success rate, thereby establishing a solid data foundation for the subsequent bibliometric network analysis.

#### 2.2.3 Processing of other fields

Beyond the issues with the CR field, Scopus bibliographic data showed significant format heterogeneity and the lack of field standardization, which impede rigorous bibliometric analysis. Existing software packages, including VOSviewer and CiteSpace, offer limited data conversion functionalities, leading to systematic formatting errors in several key fields: i. the PY field contains non-numeric characters and inconsistent formatting, often preventing recognition by CiteSpace; ii. the UT field has embedded record terminators that interfere with parsing algorithms; iii. the serial number (SN) and journal abbreviation (J9) fields are improperly merged within single records; and iv. the author affiliation (C1) field fails to properly aggregate co-authors by institution, leading to artificial inflation of inter-institutional collaboration metrics. Furthermore, the institution metadata (C3) and reprint address (RP) fields, which also pertain to author information, lack proper formatting, resulting in errors in final identification.

To resolve these systemic data quality issues, we utilized a Python-based natural language processing algorithm to standardize the integrated bibliography. This automated workflow comprised four core components: 1. a field-specific regular expression parsing engine to correct the formatting of the PY, UT, and SN fields according to WoS standards; 2. implementation of a fuzzy string matching algorithm (SequenceMatcher) with an 85% similarity threshold to identify and merge semantically equivalent institutional names; 3. intelligent parsing and restructuring of semicolon-delimited Scopus C1 strings to generate standardized C1, C3, and RP fields; and 4. automated content boundary detection and cleaning using the copyright symbol (©) as an anchor to remove residual formatting artifacts.

The standardization algorithm successfully processed complex C1 strings formatted as “Author Last Name, F. M., Institution; Author Last Name, F. M., Institution;” and converted them into the standard WoS C1 format, namely “[Author Last Name, F. M.; Author Last Name, F. M.] Institution Abbreviation.” Institutional names were abbreviated following established bibliometric conventions (e.g., University → Univ, Institute → Inst, and Department → Dept). Furthermore, while Scopus address information can be disordered, WoS favors a concise, mail-ready format, typically ordered as [Primary Institution], [Sub-unit/Department], [Street Address], [City], [State/Province], [Postal Code], and [Country]. We, therefore, optimized the address sequence to ensure that the primary institution appears first and the country appears last.

Upon the completion of automated processing, two independent researchers conducted a systematic cross-validation of the standardized bibliographic records, manually verifying author-affiliation matching, institutional consolidation, and field formatting consistency across the entire dataset. This quality assurance process required approximately 10.5 h and achieved 100% inter-rater agreement. The resulting standardized dataset containing properly formatted records provided the foundation for ensuring the integrity of the subsequent bibliometric network analysis and citation mapping.

#### 2.2.4 Entity disambiguation and thesaurus construction

The final data processing stage focused on the disambiguation of key entities—including authors, institutions, keywords, cited journals, and countries—to prepare a refined dataset for network analysis. This process involved two primary steps. The first step was preliminary consolidation. This procedure began in CiteSpace, where we generated initial visualization maps for each entity category and then exported their corresponding “Label” fields into separate CSV files. Subsequently, we executed a custom Python script to systematically identify and merge lexical variants. The script utilized a multi-faceted algorithm that combined string similarity assessment, using the difflib.SequenceMatcher ratio (with empirically optimized thresholds, such as >0.80 for journals), with a set of rule-based heuristics. To resolve matching conflicts arising from similar but distinct entities (e.g., institutional variants or journal abbreviations), we implemented a definitive hierarchical resolution protocol. The algorithm first queried a pre-built exact-match dictionary of high-frequency terms. If unmatched, it applied the SequenceMatcher. In instances of persistent ambiguity where two organizations showed high lexical similarity but distinct structural metadata, the system prioritized the entity with the most comprehensive string (i.e., containing both the primary institution and sub-unit). Any unresolved conflicts were immediately flagged for manual ‘human-in-the-loop’ verification using unique entity DOIs. The script generated a comprehensive report of suggested merge candidates, which then underwent rigorous manual review to ensure semantic consistency and accuracy. This validation process involved the examination and confirmation of similar labels over approximately 6 h.

Following validation, we compiled the confirmed rules into two software-specific thesaurus files. For CiteSpace, we created a citespace.alias file listing the canonical terms and their identified variants. Concurrently, we created a Thesaurus.txt file for VOSviewer, mapping each variant to its standardized counterpart. Furthermore, considering that some data could not be correctly identified or that invalid terms might be recognized, we also compiled a stop-word list to remove non-informative terms such as “human,” “and,” and “of.” This structured approach ensures that entity aggregation is performed dynamically and accurately across both visualization tools, thereby significantly enhancing the validity of the subsequent network analysis. These files were placed in their respective software directories, and the data were re-processed iteratively until the data were deemed clean and stable.

To address the issue where CiteSpace cannot distinguish between identical terms used in different contexts (e.g., ‘Cell’ as a keyword versus a journal title), we created distinct folders for each entity type—keywords, authors, institutions, countries, CRs, and cited authors. For each analytical run, only the relevant thesaurus file was placed in the software directory to ensure accurate disambiguation. Additionally, different bibliometric tools utilize distinct data format conventions and case-sensitivity rules; CiteSpace maintains case-sensitive entity matching, whereas VOSviewer applies case-insensitive merging. We reconciled these differences by merging the thesaurus files generated from both tools to create the final comprehensive thesaurus and stop-word lists. These master lists were then adapted for use in Bibliometrix, CiteSpace, and VOSviewer to ensure consistency across all platforms.

Notably, since this study utilizes multiple tools for keyword co-occurrence analysis, we explicitly defined our keyword field processing to ensure methodological rigor and reproducibility. Our keyword data comprised both Author Keywords (DE) and database-generated Keywords Plus (ID) to construct a comprehensive keyword co-occurrence network. This method is suitable for mapping a broad research landscape. This choice ensures high transparency and precision in our analysis, avoiding the methodological ambiguity that can arise from differing keyword sources.

#### 2.2.5 Analytical methods

To delineate the knowledge structure and evolutionary dynamics of the research domain, we conducted a multi-modal scientometric analysis. First, we quantified the longitudinal trajectory of publication output using Python to establish the field’s macroscopic growth dynamics. To probe its intellectual structure more deeply, we adopted a synergistic approach utilizing both VOSviewer (v1.6.20) and CiteSpace (v6.4. R1) (Chen, 2006), leveraging their distinct algorithmic foundations to obtain a comprehensive view. VOSviewer’s methodology, based on total link strength, was used to map static network topology, thereby identifying core actors and thematic clusters. Concurrently, CiteSpace was employed to resolve the field’s temporal evolution, applying local citation frequency counting and burst detection algorithms to track the emergence of research fronts and identify key intellectual turning points. This integrated framework enabled us to systematically analyze international collaborations, author co-authorships, and co-citation networks. To place abstract relational data in a physical context, we ultimately rendered the global geospatial distribution of scholarly output using Tableau Public and Scimago Graphica, thereby providing a geospatial dimension to complement the network analyses.

To visualize higher-order dependencies within the citation data, we utilized the general alluvial generator developed by Holmgren et al. (2023), a tool capable of handling higher-order networks such as second-order networks. This second-order model, in contrast to traditional first-order models, can capture ‘memory’ effects in citation flows. We utilized this model to construct a bibliometric alluvial diagram, aiming to reveal interdisciplinary journals that perform pivotal functions across multiple research domains. This approach allowed for more accurate identification and tracing of journal citation patterns across different disciplines, thereby constructing a temporal bibliometric map that better reflects complex citation dynamics. Furthermore, to ensure maximal consistency between the final results from CiteSpace and VOSviewer, in addition to using identical thesaurus and stop-word lists, the LBY parameter in CiteSpace was set to −1 and the k value was set as large as possible (k = 1,000) to maximize alignment with data-processing in VOSviewer.

## 3 Results

### 3.1 Analysis of the number of publications and citations

**FIGURE 2:**
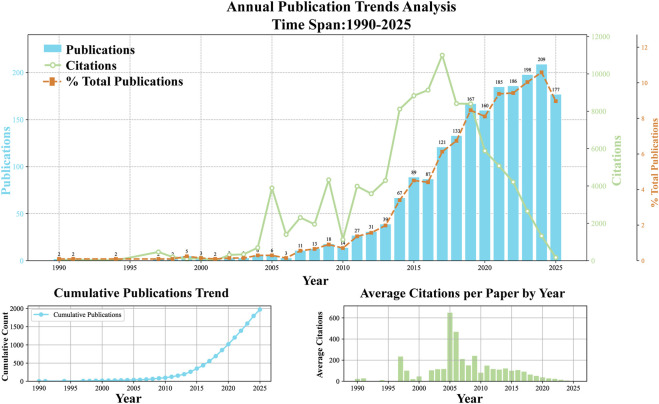
Trends in the annual publications and cited articles related to Tumor Mechanics from 1990 to 2025.

A total of 1,947 publications were included in this screening. According to Figure 2 and Table 1, a systematic bibliometric analysis was conducted on the publication volume and citation counts for the period 1976–2025. The results reveal a significant growth trend and a clear developmental trajectory over the three preceding decades. A significant temporal growth trend was observed for publication volume (R^2^ = 0.7654, *p* < 0.001), with an average annual growth rate of 6.55 publications per year. Research output, beginning with two publications in 1990, progressed through three distinct phases, namely a slow initiation phase (1990–2005), a rapid growth phase (2006–2020), and a high-output phase (2021–2025). Notably, the output peaked in 2024 with 209 publications, accounting for 10.59% of the total, which indicates that the field is currently in a highly active period of scholarly production.

**TABLE 1:** Publication and citation statistics analysis for the 1990–2025 time period.

Analysis type	Statistic	Value
Document trend regression analysis	R^2^	0.7654
	Regression rate	6.55
	*P*	<0.001
Citation trend regression analysis	R^2^	0.3634
	Regression rate	201.92
	*P*	<0.001
Correlation analysis	Pearson correlation coefficient (r)	0.4856

R^2^: Coefficient of determination.

A significant temporal trend was also observed for citation counts (R^2^ = 0.3634, *p* < 0.001), with an average annual increase of 201.92 citations. However, the temporal variability in citations is markedly higher than that of publications as reflected by the lower R^2^ value, indicating the complex and latent nature of citation practices. Notably, despite the sustained growth in publications, the average number of citations per paper has shown a declining trend, decreasing from several hundred in the early years to 6.34 in 2024. This likely reflects a dilution effect, whereby literature growth outpaces citation accumulation. Pearson correlation analysis indicates a moderate positive correlation between publication volume and citation counts (*r* = 0.4856), which aligns with fundamental bibliometric principles. However, the correlation strength is modest, indicating that factors beyond publication volume—such as paper quality, shifts in research hotspots, and journal impact—exert considerable influence on citation counts.

Based on the cumulative publication curve and annual output patterns, the research domain has transitioned from an emerging discipline to a mature stage of development. Over 32 years, the field has produced 1,947 papers and accumulated 102,456 citations, demonstrating substantial academic impact and sustained research vitality. The current high level of productivity and relatively stable growth indicate that the field may be approaching or has reached a plateau phase.

### 3.2 Analysis of the contributions of prolific and co-cited authors

This study presents a systematic bibliometric assessment of the authors who have contributed to the field. Among the scholars analyzed, Table 2 highlights the 10 most prolific authors, with Valerie M. Weaver leading at 28 publications, followed by Cynthia A. Reinhart-King (26 publications) and Triantafyllos Stylianopoulos (23 publications). A cross-comparison with the list of corresponding authors reveals a critical characteristic: these highly productive scholars are also the core leaders in this domain (Table 3). For instance, Weaver, Reinhart-King, Stylianopoulos, Youhua Tan, and Yiyao Liu are not only among the most prolific authors but also rank as the top corresponding authors. This indicates that they not only possess high individual research productivity but also direct the academic trajectory of their respective research groups. Citation metrics further substantiate the scholarly influence of these core researchers; the top three authors each have more than 2,000 total citation counts, reflecting the extensive recognition of their work. The h-index, a composite measure of productivity and impact, also verifies their significant contributions (e.g., Weaver’s h-index of 24).

**TABLE 2:** Top 10 productive authors and their representative article.

Rank	Author	Country	Documents	Citations	Average citation	H-index	Representative work
1	Weaver, Valerie M	United States	28	5,468	195.29	24	The extracellular matrix modulates the metastatic journey
2	Reinhart-King, Cynthia A	United States	26	2,224	85.54	20	Targeting extracellular matrix stiffness to attenuate disease: From molecular mechanisms to clinical trials
3	Stylianopoulos, Triantafyllos	Cyprus	23	3,091	134.39	16	Angiotensin inhibition enhances drug delivery and potentiates chemotherapy by decompressing tumor blood vessels
4	Tan, Youhua	China	18	587	32.61	12	Matrix softness regulates plasticity of tumor-repopulating cells via H3K9 demethylation and Sox2 expression
5	Mierke, Claudia T	Germany	16	1,002	62.62	11	The matrix environmental and cell mechanical properties regulate cell migration and contribute to the invasive phenotype of cancer cells
6	Liu, Yiyao	China	16	700	43.75	12	Mechanosensitive caveolin-1 activation-induced PI3K/AKT/mTOR signaling pathway promotes breast cancer motility, invadopodia formation, and metastasis in vivo
7	Song, Guanbin	China	15	329	21.93	10	Stiffer matrix accelerates migration of hepatocellular carcinoma cells through enhanced aerobic glycolysis via the MAPK-YAP signaling
8	Cui, Jiefeng	China	15	755	50.33	11	Higher matrix stiffness as an independent initiator triggers epithelial–mesenchymal transition and facilitates HCC metastasis
9	Keely, Patricia J	USA	13	2,273	174.85	13	Biomechanical remodeling of the microenvironment by stromal caveolin-1 favors tumor invasion and metastasis
10	Chen, Rongxin	China	13	716	55.08	10	Higher matrix stiffness as an independent initiator triggers epithelial–mesenchymal transition and facilitates HCC metastasis

**TABLE 3:** Top 10 corresponding authors and their representative article.

Rank	Corresponding author	Documents	Total citations	First year	Last year	Representative work
1	Reinhart-King CA	18	1,517	2012	2025	Targeting extracellular matrix stiffness to attenuate disease: From molecular mechanisms to clinical trials
2	Weaver V.M.	28	5,949	2009	2025	Tensional homeostasis and the malignant phenotype
3	Stylianopoulos T.	12	2,662	2014	2025	Hippo pathway in organ size control, tissue homeostasis, and cancer SO cell
4	Tan YH	12	267	2019	2025	Mechanics and actomyosin-dependent survival/chemoresistance of suspended tumor cells in shear flow
5	Mierke CT	11	606	2014	2025	The matrix environmental and cell mechanical properties regulate cell migration and contribute to the invasive phenotype of cancer cells
6	Weihs D	9	165	2012	2023	Intracellular mechanics and activity of breast cancer cells correlate with metastatic potential
7	Song GB	9	262	2018	2025	Stiffer matrix accelerates migration of hepatocellular carcinoma cells through enhanced aerobic glycolysis via the MAPK-YAP signaling
8	Papavassiliou AG	8	112	2017	2025	Recent advances in mechanobiology of osteosarcoma
9	Nelson CM	7	198	2012	2021	Host epithelial geometry regulates breast cancer cell invasiveness
10	Liu YY	7	384	2014	2022	Mechanosensitive caveolin-1 activation-induced PI3K/AKT/mTOR signaling pathway promotes breast cancer motility, invadopodia formation, and metastasis *in vivo*

**FIGURE 3:**
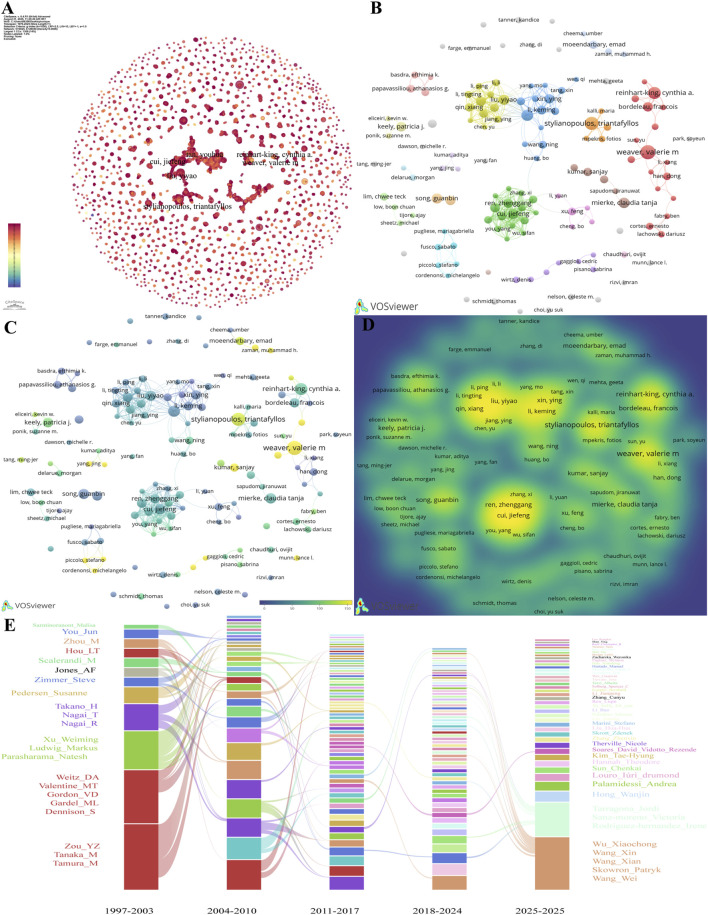
Author contributions to publications in tumor mechanics. **(A)** Network visualization of co-authorship, which was generated using CiteSpace. **(B)** Network visualization of co-authorship, which was generated using VOSviewer. **(C)** Overlay visualization of co-authorship, which was generated using VOSviewer. **(D)** Density visualization of co-authorship, which was generated using VOSviewer. **(E)** Alluvial plot of the temporal evolution of contributions by co-authorship from 1997 to 2025.

Analysis of the author collaboration network (Figures 3A,B) shows that the research community is not uniformly distributed in a single integrated network but is organized into several cohesive yet relatively independent collaborative clusters. Within the collaboration networks centered on Jiefeng Cui and Youhua Tan (green cluster) and Yiyao Liu (blue cluster), the nodes representing these core members exhibit a yellow–green color, indicative of high citation impact. This confirms that they are not only collaborative hubs within their respective research teams but also play a pivotal academic leadership role, with their research outputs being the primary source of the team’s influence. Furthermore, in the overlap and density views (Figures 3C,D), the nodes for Valerie M. Weaver, Cynthia A. Reinhart-King, and Triantafyllos Stylianopoulos all display the most vibrant yellow, indicating that their published papers have received exceptionally high average citation counts. These visualizations also enable the identification of potential high-impact experts. Certain scholars in the graph have small node sizes (i.e., relatively few publications) but bright yellow node colors. Examples include Stefano Piccolo, Michelangelo Cordenonsi, Lance L. Munn, Fan Yang, and Muhammad H. Zaman. This indicates that they may have contributed a small number of pioneering or “milestone” papers with high citation rates, representing significant domain-specific expertise or emerging research forces that should not be overlooked. Based on the temporal impact graph in Figure 3E, the research landscape in tumor mechanics has undergone substantial changes since 1997. The influence of groups led by Y.Z. Zou and H. Takano has diminished over time. Beginning in the 2011–2017 period, new research directions and teams have emerged, with the group represented by Xiaochong Wu contributing to the rise of this new focus. Additionally, some teams, such as that of D.A. Weitz, have demonstrated stable and continuous contributions.

**FIGURE 4:**
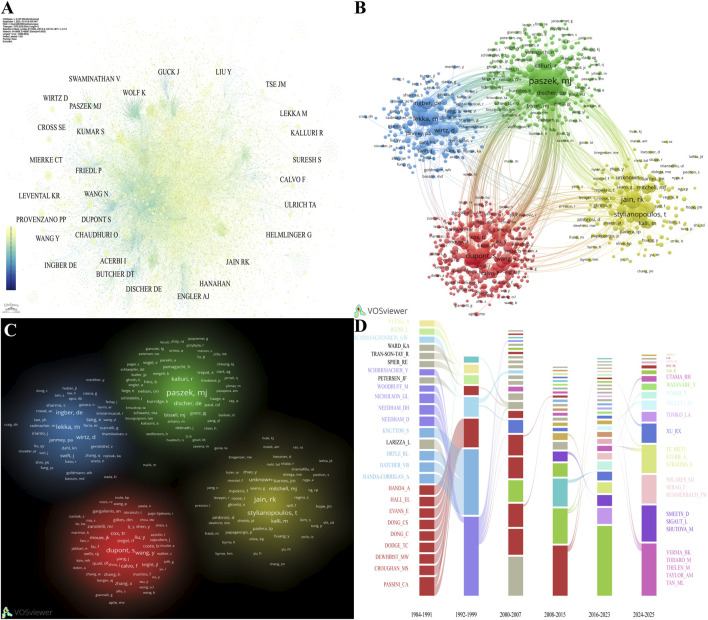
Contributions of co-cited authors to publications in tumor mechanics. **(A)** Network visualization of co-cited authors, which was generated using CiteSpace. **(B)** Network visualization of co-cited authors, which was generated using VOSviewer. **(C)** Density visualization of co-cited authors, which was generated using VOSviewer. **(D)** Alluvial plot of the temporal evolution of contributions by co-cited authors’ contributions from 1984 to 2025.

Co-citation analysis shows that M.J. Paszek is the most frequently co-cited author (576 occurrences), followed by P.P. Provenzano (494) and K.R. Levental (437) (Table 4), indicating that their work has had a foundational influence on the intellectual paradigms of the field. A deeper analysis of the co-citation network visualization (Figures 4A–C) demonstrates that the field’s knowledge base is not dominated by a single theory but is built upon four primary interconnected theoretical branches, forming distinct clusters. The cluster centered around M.J Paszek primarily focuses on the biophysical interactions between cells and the microenvironment. The group led by R.K. Jain and T. Stylianopoulos concentrates on the mechanical properties of the tumor microenvironment and drug delivery. The cluster represented by P. Friedl and M. Lekka is oriented toward the mechanical mechanisms of cell invasion and migration. Finally, the team with S. Dupont as a key node is likely associated with the study of critical signaling pathways in mechanotransduction. The temporal impact graph (Figure 4D) shows that M.S. Croughan and C.A. Passini were the earliest cornerstones of the field, a finding that corresponds to data from the cited references section. However, as time progressed, the academic landscape experienced another significant shift during 2000–2015, closely mirroring the changes observed among collaborating authors. From 2016 to the present, the contributing areas of cited authors have become increasingly diversified. Nevertheless, the light-blue group of R.L. Jirtle shows remarkable persistence; although never the most dominant force after the initial period, its influence is sustained throughout the entire timeline.

**TABLE 4:** Top 10 co-cited authors and their representative article.

Rank	Co-cited author	Country	Citation frequency	First cited year	Last cited year	Representative work
1	Paszek, MJ	USA	576	2004	2014	Tensional homeostasis and the malignant phenotype
2	Provenzano, PP	USA	494	2003	2013	Matrix density-induced mechanoregulation of breast cell phenotype, signaling, and gene expression through a FAK–ERK linkage
3	Levental, KR	USA	437	2009	2010	Matrix crosslinking forces tumor progression by enhancing integrin signaling
4	Jain, RK	USA	383	1984	2021	The role of mechanical forces in tumor growth and therapy
5	Stylianopoulos, T	USA	326	2007	2018	Causes, consequences, and remedies for growth-induced solid stress in murine and human tumors
6	Friedl, P	USA	319	1900	2017	Tumor-cell invasion and migration: diversity and escape mechanisms
7	Dupont, S	Italy	307	2011	2022	Role of YAP/TAZ in mechanotransduction
8	Hanahan, D	Swiss	274	1996	2022	Hallmarks of cancer: the next-generation
9	Mierke, Claudia Tanja	Germany	273	2008	2024	The matrix environmental and cell mechanical properties regulate cell migration and contribute to the invasive phenotype of cancer cells
10	Lekka, M	Poland	261	1999	2022	Elasticity of normal and cancerous human bladder cells studied by scanning force microscopy

### 3.3 Analysis of the cited journal contributions

A detailed analysis of the journal citation landscape within a scholarly field is crucial for understanding its knowledge structure, core topics, and evolutionary pathways. By utilizing a comprehensive suite of bibliometric methods—including multi-dimensional visualization of core journal communities, co-citation network structures, and interdisciplinary knowledge flows—this study aims to construct a panoramic map of the field’s intellectual ecosystem.

**FIGURE 5:**
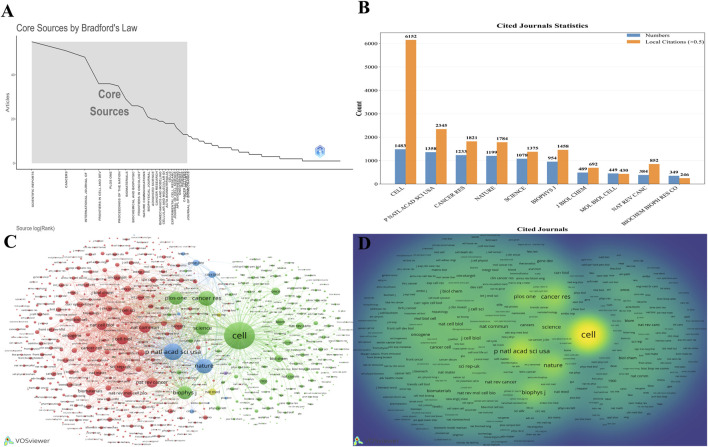
Analysis of the academic journals. **(A)** Identification of core source journals according to Bradford’s Law. **(B)** Citation frequency of the top 10 most-cited journals. **(C)** Network visualization of journal co-citation. **(D)** Density visualization of journal co-citation.

The knowledge base of this domain is supported by a core set of academic journals. A distribution map of core journals based on Bradford’s Law (Figure 5A) demonstrates that a small number of journals publish a large volume of the key literature in the field. Quantitative analysis of the most frequently cited journals further specifies the composition of this core group. *Cell* ranks first with 1,483 citations, followed by the *Proceedings of the National Academy of Sciences of the United States of America* (PNAS), *Cancer Research*, *Nature*, and *Science*. Together, these journals constitute the authoritative sources of knowledge in this domain (Figure 5B). This citation pattern is visually corroborated in the journal co-citation network knowledge map, where these journals, represented by large nodes, form the central backbone of the entire network, underscoring their undisputed academic influence.

The co-citation network not only identifies core journals but also reveals the intrinsic relationships between different research directions within the field (Figures 5C,D). The map clearly displays multiple color-coded journal clusters, representing distinct thematic areas; for example, the cluster centered on *Cancer Research* evidently focuses on oncology. These clusters are not entirely isolated but are interconnected by journals such as *Cell* and *Nature*, which possess both high-impact and interdisciplinary characteristics. These journals function as knowledge hubs, facilitating the exchange and integration of ideas across different research paradigms. Notably, influence is not solely determined by the citation frequency; network structure metrics offer richer insights. For instance, although a journal such as the *Biology of Reproduction* is not among the most cited, its degree centrality is as high as 194, indicating that it occupies a critical bridging position in the knowledge dissemination network and serves as a vital node for the efficient flow of information.

**FIGURE 6:**
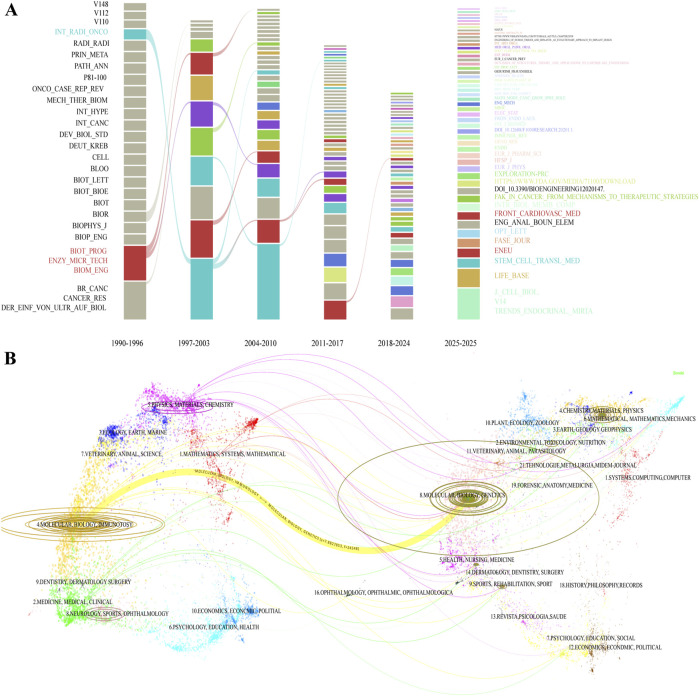
Evolutionary analysis of the cited journals. **(A)** Alluvial plot of the temporal evolution of cited journals from 1990 to 2025. **(B)** Dual-map overlay illustrating the citation flow among journal disciplines.

The dynamic evolution of knowledge in this field is further elucidated through a time-series analysis of journal influence (1990–2024) and a dual-map overlay of the journal landscape (Figure 6). The former illustrates the sustained influence of core journals and the shifting trajectory of research hotspots over time. The temporal evolution of journal publication patterns exhibits distinct phases: the early stage (1990–2003) was concentrated in traditional biology journals, the period from 2004 to 2017 saw the rise of interdisciplinary journals, and the years 2018–2025 have been characterized by the rapid development of emerging cross-disciplinary journals. The ascent of new journals, such as *Nature Biomedical Engineering* and *Science Advances*, reflects the field’s trend toward applied technology.

The dual-map overlay depicts the trajectory of knowledge inheritance and innovation at the disciplinary level. The cluster of cited journals on the left represents the knowledge base, while the cluster of citing journals on the right signifies the research front. The analysis shows that the field’s knowledge base is primarily rooted in disciplines such as molecular biology, immunology, medicine, and physical chemistry. The research front, in contrast, is expanding toward molecular genetics, health sciences, and environmental sciences. A prominent, thick yellow citation path clearly delineates a strong flow of knowledge from the “Molecular Biology/Immunology” cluster on the left to the “Molecular Biology/Genetics” cluster on the right. This core knowledge trajectory powerfully explains why molecular biology journals, led by *Cell*, hold a dominant position in the field; their research paradigms and findings constitute the central driving force behind the development of the entire domain. Concurrently, the presence of multiple secondary and cross-disciplinary paths indicates that while the field maintains a strong core trajectory, it also exhibits a trend of multi-disciplinary knowledge permeation and integration, thereby collectively constructing a complex knowledge network that is both stable and vibrant.

### 3.4 Analysis of institutional contributions

**FIGURE 7:**
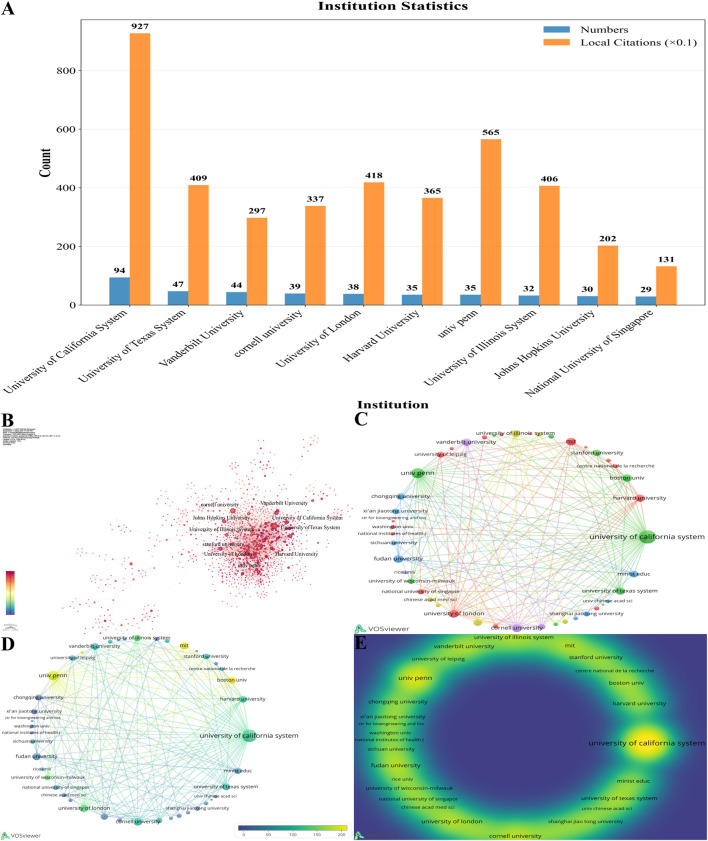
Institutional contributions and collaborations. **(A)** Bar chart analysis of publication output from the top 10 institutions. **(B)** Network visualization of inter-institutional collaboration, which was generated using CiteSpace. **(C)** Network visualization of inter-institutional collaboration, which was generated using VOSviewer. **(D)** Overlay visualization of inter-institutional collaboration, which was generated using VOSviewer. **(E)** Density visualization of inter-institutional collaboration.

A bibliometric analysis of the research institutions in this field reveals a highly centralized and hierarchical structure in global academic output and collaboration networks, with a few top-tier institutions forming the core of knowledge production and dissemination (Figure 7). In terms of publication volume, the University of California system holds an unequivocal leadership position with 90 publications, making it the most prolific research entity in the field. It is followed by the University of Texas System (40 publications), Cornell University (37), the University of London (36), and the University of Pennsylvania (Univ Penn) and Harvard University (35 each). These institutions collectively constitute the top echelon of high-output organizations (Figure 7A).

At the level of academic impact, citation analysis reveals a more complex relationship between publication volume and influence. The University of California System not only leads in output but also ranks first in citation frequency, demonstrating that its research possesses both breadth and depth. However, some institutions demonstrate a disproportionately high citation-to-publication ratio, with the University of Pennsylvania being particularly noteworthy. Although it produced 35 publications, its citation impact ranks second overall, indicating that its research output is of extraordinary quality and leadership, with a higher likelihood of producing seminal contributions to the field.

Institutional collaboration network analysis further elucidates this knowledge-exchange landscape, which is dominated by core institutions (Figures 7B–E). Various metrics for identifying central nodes, such as degree centrality, point to the same leaders. The University of California System, with a degree centrality of 143, is the most central hub in the entire network, followed by the University of Texas System (103) and the University of London (100). This indicates that the most prolific institutions are also the most extensively connected and active nodes in the collaboration network. Notably, the high degree centrality of the University of London highlights its crucial role as a bridge connecting North American and European research networks. Furthermore, some European institutions, such as the Consiglio Nazionale delle Ricerche (Italy) and the CNRS—National Institute for Biology (France), possess a high degree of centrality within the network despite not being ranked among the top ten for publication volume, signifying their indispensable roles as regional collaboration centers. Concurrently, the structural variation metric (Sigma) for all these core nodes is 1.00, further confirming their critical status in maintaining the network’s structural stability and information flow.

**FIGURE 8:**
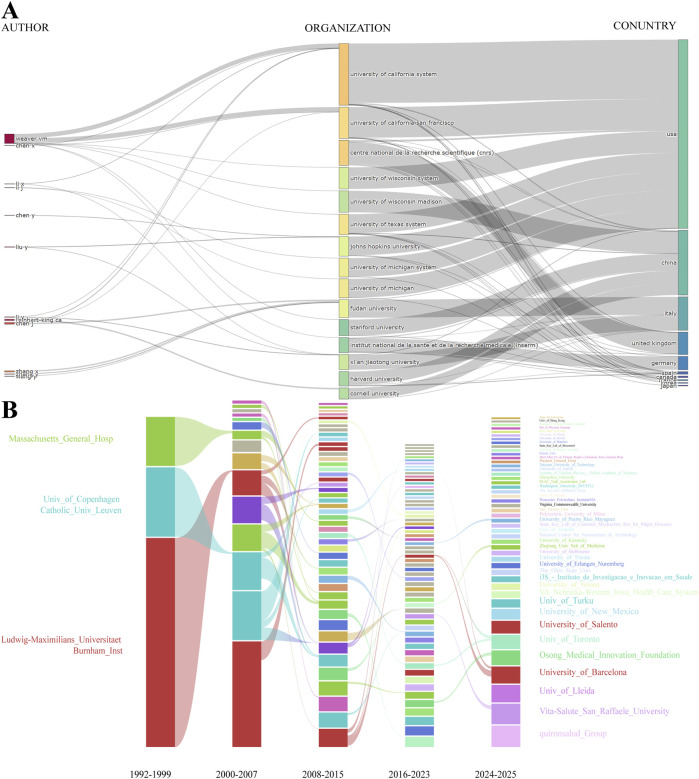
Evolutionary analysis of authors, institutions, and countries. **(A)** Sankey diagram illustrating the evolutionary links among authors, institutions, and countries. **(B)** Alluvial plot of the temporal evolution of institutional contributions from 1992 to 2025.

An impact analysis based on the country of author affiliations (Figure 8A) shows the dominant position of institutions from the United States. The University of California system ranks first with a total link strength of 1,033, followed by the University of Pennsylvania is second with 632. Among Chinese institutions, Fudan University performs best with a total link strength of 369, while Huazhong University of Science and Technology (147) and Shanghai Jiao Tong University (177) also demonstrate considerable international collaboration capacity. The density analysis indicates close collaboration between American and European institutions, whereas Asian institutions are relatively more independent, indicating that the breadth of their international collaboration could be enhanced.

The temporal evolution analysis from the alluvial diagram reveals phased changes in the institutional contribution landscape (Figure 8B). The early stages were primarily dominated by traditional research institutions from Europe and the United States. In recent years, emerging institutions and research centers from developing countries have progressively entered the field, signaling the potential for a more balanced global landscape of institutional contribution in the future.

### 3.5 Analysis of country contributions

**FIGURE 9:**
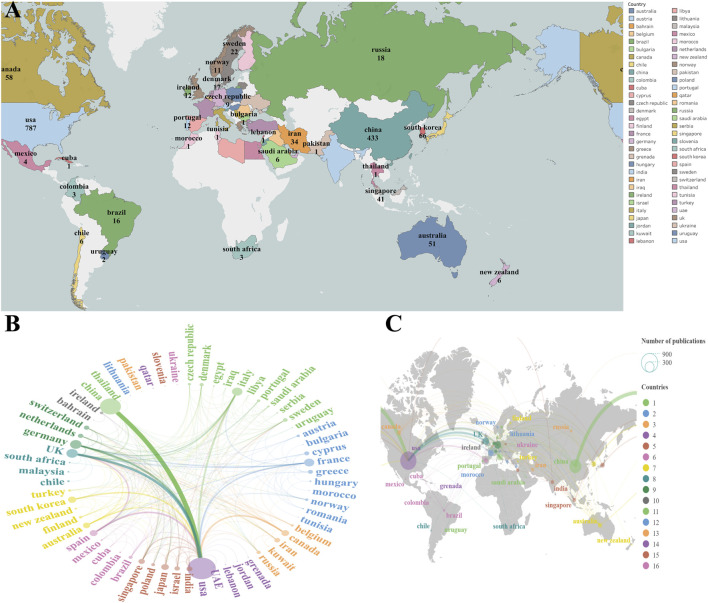
National contributions and collaborations. **(A)** Geospatial visualization of publication output by country. **(B)** Network analysis of the strength of international collaborations. **(C)** Geospatial overlay illustrating the network strength of international collaborations.

The scholarly output in this field demonstrates pronounced geographic clustering, with a minority of countries contributing the majority of academic achievements and impact. The United States (US) and China constitute the two poles of this domain in terms of both publication volume and scholarly influence. The US is the undisputed leader, ranking first in both the number of publications and total citations. China has emerged as a key force, firmly securing the second position in both output and impact. These two leaders are followed by traditional European scientific powerhouses, including Germany, the United Kingdom, and Italy (Figure 9A).

The international collaboration network exhibits a core–periphery topology centered on the US. Centrality analysis quantifies the network role of each country: the US and the UK not only possess the most extensive collaborative reach (high degree centrality) but also serve as indispensable bridges in knowledge dissemination (highest betweenness centrality). A particularly notable phenomenon is the significant disparity between China’s research output and its network position. Despite its substantial publication volume, its network centrality is relatively low, especially in its limited role as a collaborative intermediary. This indicates that its integration and leadership within the global collaborative network have room for improvement, presenting a characteristic of “high output, weak connectivity” (Figures 9B,C). It should be noted that the “weak connectivity” here refers to international collaborations. Based on co-author contribution data (Figures 3, 4), it is evident that Chinese collaborators tend to cooperate frequently with one another, yet their international influence remains comparatively weaker. Therefore, the national collaboration patterns are congruent with the author-level collaboration data.

**FIGURE 10:**
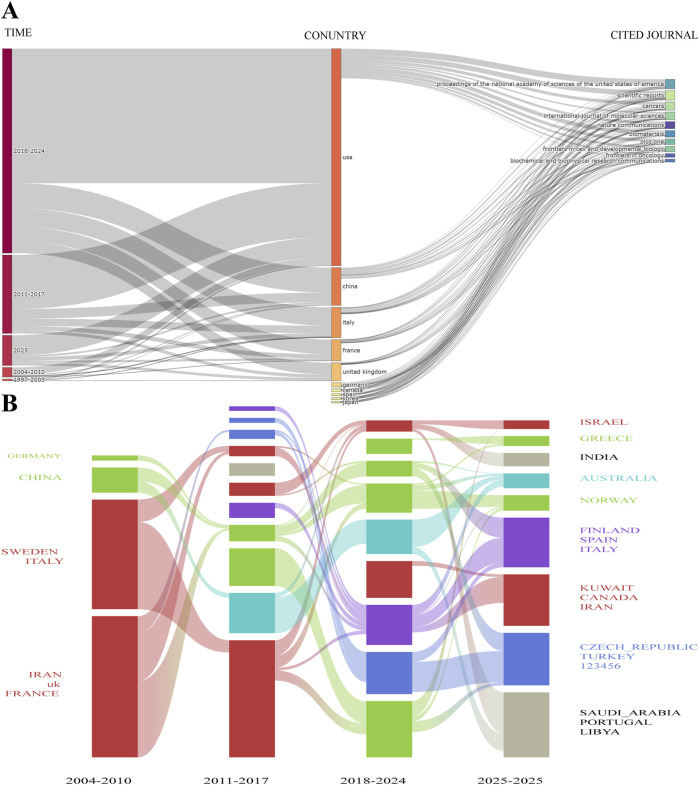
Spatiotemporal and source analysis of national publications. **(A)** Sankey diagram illustrating the relationships among time, country, and journal. **(B)** Alluvial plot of the temporal evolution of national publication output from 2004 to 2025.

From a temporal perspective, the landscape of international collaboration has undergone profound phased transformations (Figure 10). Between 2004 and 2010, the network was dominated by developed nations in Europe and North America, forming a relatively closed “elite club.” Between 2011 and 2017, with the explosive growth of its scientific research capabilities, China’s position within the network enhanced significantly, and it began to engage more deeply in international collaborations, thereby challenging the previous US–European dominance. From 2018 to the present, the collaboration model has further evolved toward diversification and polycentricity, with an increasing number of research entities integrating into the global research system. This marks a growing globalization and openness in the research activities of the field. The geospatial distribution visualization is highly consistent with the network analysis, clearly delineating three major research clusters centered in North America (led by the US), Europe (led by Germany, the UK, and France), and Asia (led by China). This geographic clustering effect profoundly reflects the dependence of frontier science on advanced research infrastructure and resources, which in turn shapes the current distribution of global scientific strength.

### 3.6 Analysis of highly cited references

**FIGURE 11:**
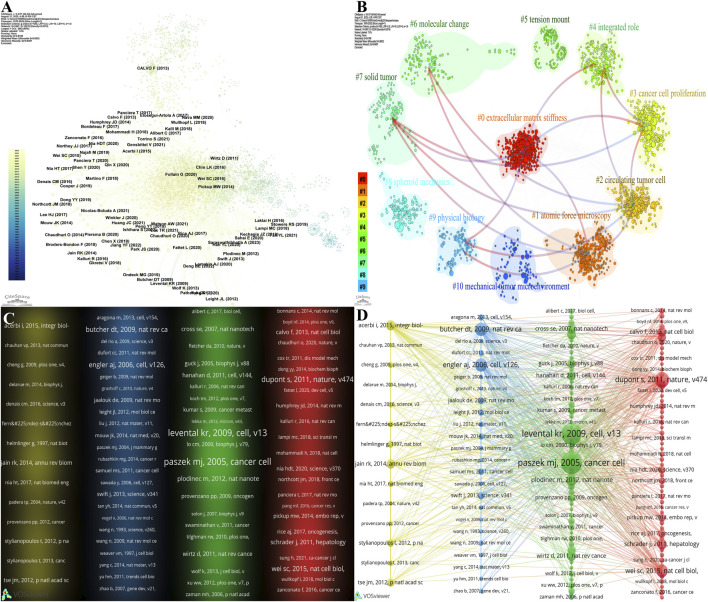
Analysis of cited references. **(A)** Network visualization of cited references, which was generated using CiteSpace. **(B)** Cluster network of cited references, which was generated using CiteSpace. **(C)** Density visualization of reference co-citation, which was generated using VOSviewer. **(D)** Network visualization of reference co-citation, which was generated using VOSviewer.

Through a bibliometric analysis of the literature on the physical tumor microenvironment, we can systematically delineate the field’s knowledge structure, evolutionary path, and research hotspots. Overall, the field has formed a well-structured and highly cohesive knowledge system centered on the main axis of “mechanical microenvironment–cell behavior–tumor progression” (Figure 11). Research by Paszek et al. (2005) (*Cancer Cell*), Sacks et al. (2018), Levental et al. (2009) (*Cell*), and Engler et al. (2006) (*Cell*), collectively, constitutes the “theoretical core” of the domain (Figure 11). Their foundational status is corroborated by multi-dimensional data: they are not only the most highly cited publications but also the most structurally important central nodes in the co-citation network. These pioneering studies demonstrated that the physical properties of the ECM, particularly matrix stiffness, serve as key regulators of malignant cellular phenotypes. Together, they form the earliest core research cluster in the field. Furthermore, the discovery by Dupont et al. (2011) (*Nature*) of YAP/TAZ as critical mechanotransduction molecules provided key molecular evidence for understanding the transmission of mechanical signals. The core literature is primarily published in top-tier journals such as *Cell*, *Nature*, and *Cancer Cell*; for example, the work by Engler et al. (2006) has been cited 11,864 times globally, indicating significant cross-disciplinary impact (Table 5).

**TABLE 5:** Top 10 most-cited references in the field of tumor mechanics.

Rank	Year	First author	Title	Source	Global citations	Local citations in dataset
1	2005	Matthew J Paszek	Tensional homeostasis and the malignant phenotype	Cancer cell	3,286	320
2	2009	Kandice R Levental	Matrix crosslinking forces tumor progression by enhancing integrin signaling	Cell	3,330	306
3	2011	Sirio Dupont	Role of YAP/TAZ in mechanotransduction	Nature	4,990	193
4	2006	Adam J Engler	Matrix elasticity directs stem-cell lineage specification	Cell	11,864	156
5	2009	Darci T Butcher	A tense situation: forcing tumor progression	Nature Reviews Cancer	1,743	156
6	2015	Spencer C Wei	Matrix stiffness drives epithelial–mesenchymal transition and tumor metastasis through a TWIST1-G3BP2 mechanotransduction pathway	Nature Cell Biology	827	145
7	2012	Marija Plodinec	The nanomechanical signature of breast cancer	Nature Nanotechnology	994	130
8	2011	Jörg Schrader	Matrix stiffness modulates proliferation, chemotherapeutic response, and dormancy in hepatocellular carcinoma cells	Hepatology	658	112
9	2007	Sarah E Cross	Nanomechanical analysis of cells from cancer patients	Nature Nanotechnology	1,711	108
10	2015	I Acerbi	Human breast cancer invasion and aggression correlates with ECM stiffening and immune cell infiltration	Integrative Biology (Camb)	944	108

**FIGURE 12:**
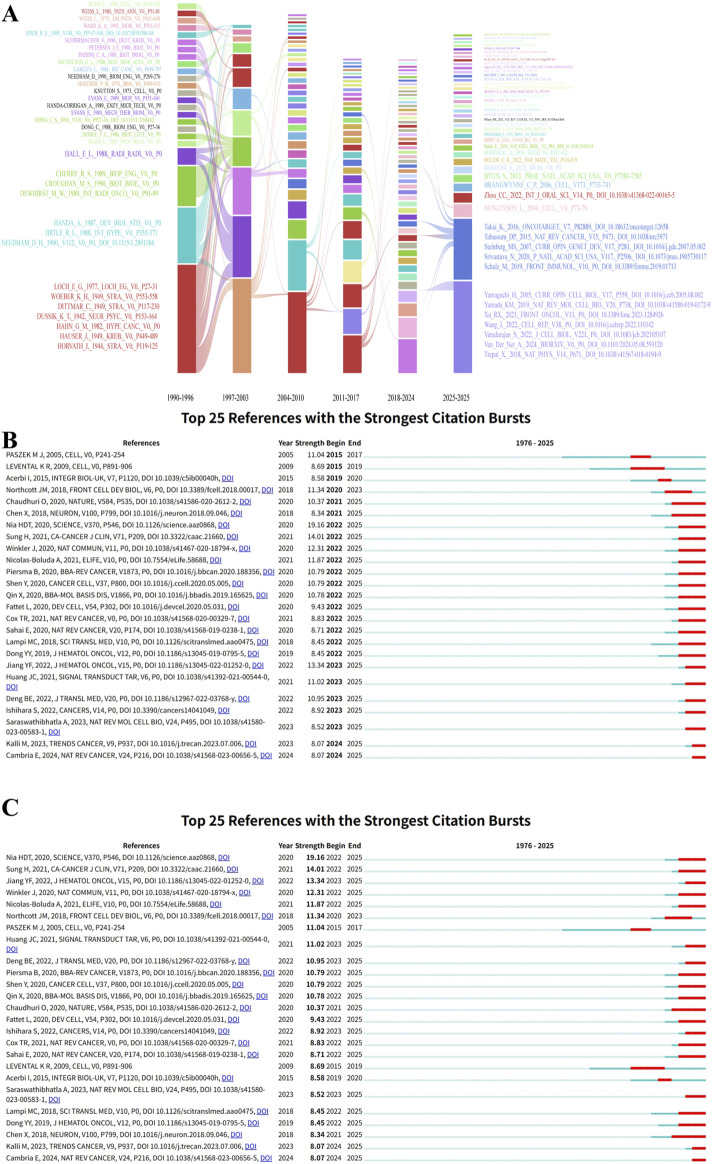
Temporal dynamics of cited references. **(A)** Alluvial plot showing the temporal evolution of cited references from 1990 to 2025. **(B)** The 25 references with the highest citation burst strength ordered chronologically. **(C)** The 25 references with the highest citation burst strength ordered by strength.

Building upon this core intellectual foundation, the research themes within the field have continuously diverged and converged over time, forming distinct research clusters whose evolving average publication years outline a clear disciplinary trajectory (Figures 11B, 12). First, methodological innovations, represented by “atomic force microscopy” (cluster #1), initiated the quantitative study of the mechanical properties of cells and tissues (Lekka et al., 1999). Second, the exploration of core mechanisms has deepened, evolving from an early focus on the link between “matrix stiffness” and “cancer cell proliferation” (cluster #3) to the recent, largest cluster of “extracellular matrix stiffness” (cluster #0), reflecting a shift toward more complex interaction mechanisms. Finally, the research perspective has expanded to clinical questions (Dong Y. et al., 2024), with the emergence of clusters such as “solid tumor” (cluster #7) (Gajda et al., 2025), “circulating tumor cells” (cluster #2) (Qiu et al., 2024), and “spheroid mechanics” (cluster #8) (Rodrigues et al., 2024), which signals a move from two-dimensional systems to three-dimensional models and clinical applications that more closely mimic *in vivo* conditions (Rauner et al., 2025).

Cross-validation between citation burst analysis and cluster analysis has precisely identified the most groundbreaking research fronts. The literature with the highest citation burst strength is highly concentrated within the newest and fastest-growing frontier clusters. For instance, burst articles by Nia et al. (2020) (*Science*) and Chaudhuri O. et al. (2020) (*Nature*) are core members of the “spheroid mechanics” (cluster #8) cluster, focusing on simulating complex *in vivo* mechanical environments. Similarly, burst articles such as Winkler et al. (2020) (*Nature Communications*) belong to the “extracellular matrix stiffness” (cluster #0) cluster, indicating that the re-exploration of how ECM stiffness precisely regulates multicellular interactions within the tumor microenvironment is a current core research hotspot.

In summary, the interdisciplinary field of the physical tumor microenvironment has evolved from its early phase of establishing the core theory of “mechanics drives cancer” to a new stage. This current phase involves the integrated use of advanced bioengineering technologies and 3D models to deeply analyze the complex interplay between the multi-scale mechanical microenvironment and tumor heterogeneity, immunity, and therapeutic response. Future research will increasingly focus on the clinical translation of mechanobiological mechanisms, thereby providing a scientific basis for the development of novel diagnostic strategies and “mechanomedicine.”

### 3.7 Keyword analysis

**FIGURE 13:**
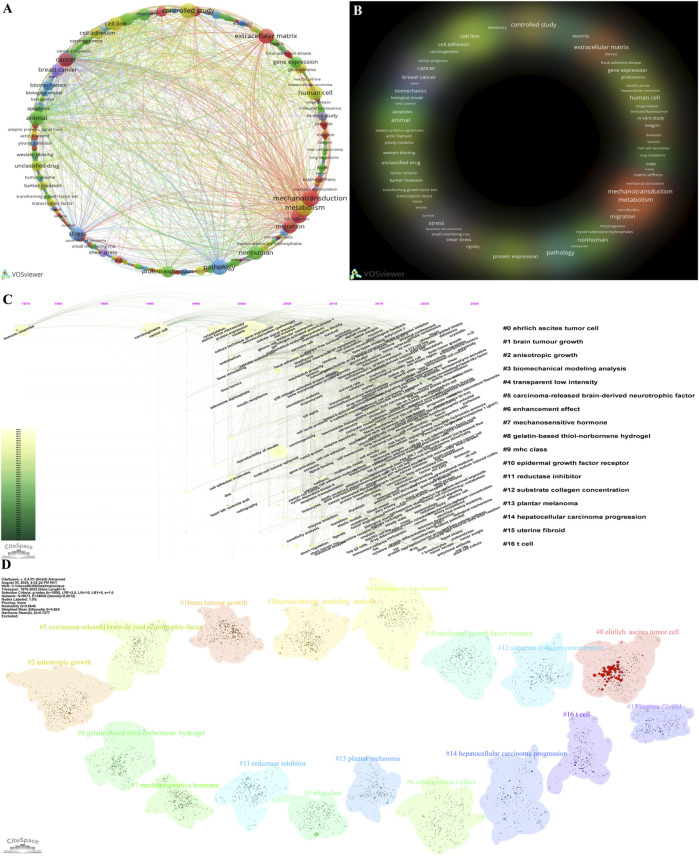
Keyword analysis. **(A)** Network visualization of keyword co-occurrence with a circular layout, which was generated using VOSviewer. **(B)** Density visualization of keyword hotspots, which was generated using VOSviewer. **(C)** Timeline of keywords from 1976 to 2025, which was generated using CiteSpace. **(D)** Cluster network of keywords, which was generated using CiteSpace.

To systematically deconstruct the intellectual core, evolutionary trajectory, and thematic ecosystem of this research domain, we conducted a multi-dimensional, multi-level bibliometric analysis of keywords. The results (Figure 13) show that “mechanotransduction,” “extracellular matrix,” and “cancer” rank among the top three keywords not only in frequency of occurrence but also in the citation counts of their associated literature. This provides strong evidence that these three concepts form the most central and fundamental research vocabulary of the field. The keyword co-occurrence network map further corroborates this finding; these high-frequency keywords act as core hub nodes in the network, closely connecting multiple important research sub-clusters related to “tumor metastasis,” “microenvironment,” and “cell migration.” This structure delineates a macroscopic knowledge landscape centered on the core scientific question of how the physical microenvironment regulates cancer biology through mechanotransduction (Liu et al., 2020).

**FIGURE 14:**
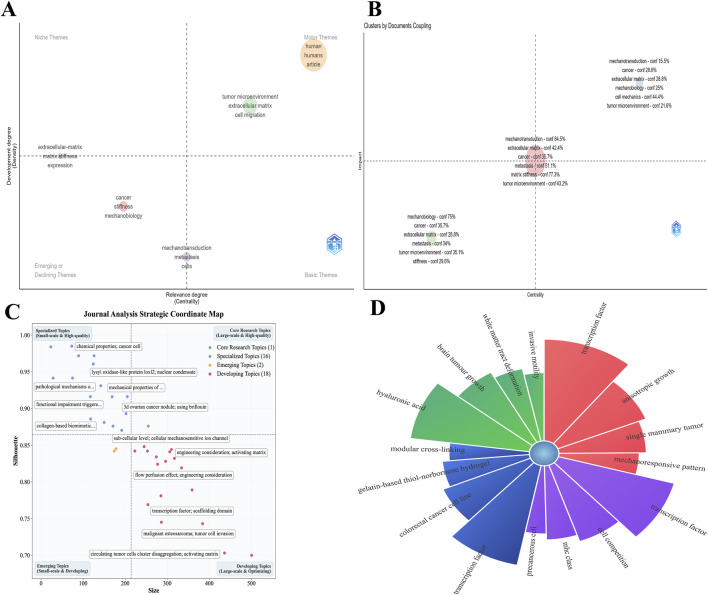
Strategic analysis of keywords. **(A)** Strategic coordinate diagram of keywords. **(B)** Strategic diagram illustrating keyword clusters. **(C)** Quality metrics for keyword analysis. **(D)** The top four keywords by strength within each cluster.

A strategic diagram analysis provides an ecological positioning for these themes (Figure 14A). The cluster comprising terms such as “mechanotransduction” and “cancer” is identified as a foundational theme, characterized by high centrality and relatively low development. This indicates that these concepts, while serving as the common theoretical bedrock connecting the majority of research, are conceptually mature and function as intellectual sources for current explorations rather than as rapidly emerging hotspots. In contrast, a theme represented by “matrix stiffness” and “fibroblast activation” is located in an emerging or peripheral zone, indicating its high potential as a future breakthrough direction (Santos and Lagares, 2018). Notably, the motor theme quadrant, which would contain topics driving the entire field forward, is vacant. This reveals a somewhat diffuse current knowledge structure, which lacks a core engine that possesses both high centrality and high development.

Coupled clustering and thematic quality diagnostics offer deeper insights into the analysis of the internal composition of this “diffuse” structure (Figures 14B,C). The analysis confirms the existence of a vast intellectual core that closely integrates foundational concepts such as “mechanotransduction” with frontier biological processes such as “tumor metastasis” and “tumor microenvironment,” forming the central pillar of the domain’s research. Simultaneously, an emerging perspective identified by “mechanobiology” and “stiffness” is taking shape; although not yet fully integrated into the mainstream framework, it represents a future incubation direction for the field. The thematic quality diagnosis more incisively reveals a ‘multi-point bloom’ ecosystem: the research landscape is not monopolized by a few large-scale, highly mature flagship topics (Figure 14D). Instead, it is predominantly driven by a multitude of developing themes [e.g., “circulating tumor cell cluster dissociation” (Ezeobidi and Truszkowska, 2025)] and specialized themes [e.g., “collagen-based biomimetic materials” (Cambi and Ventre, 2022)]. This finding logically corroborates the absence of a motor theme, indicating that the current vitality and innovation in the field are propelled by these highly active boundary-expanding developing themes in conjunction with numerous “niche” specialized topics.

**FIGURE 15:**
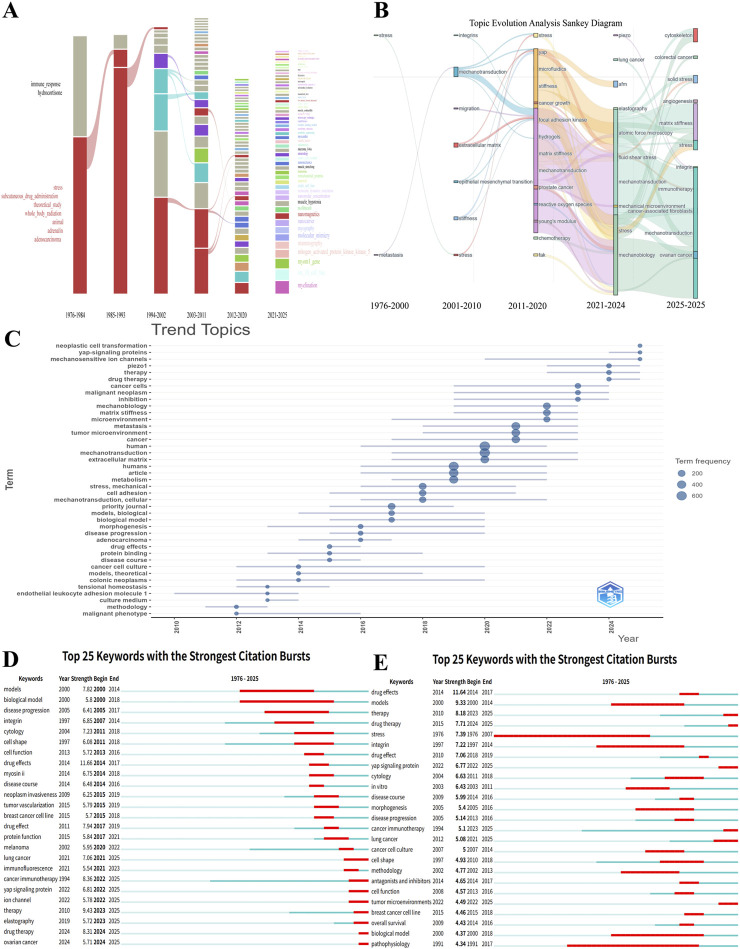
Temporal dynamics of keywords. **(A)** Alluvial plot illustrating the temporal evolution of keywords from 1976 to 2025. **(B)** Sankey diagram of keyword temporal evolution. **(C)** Timeline of keyword trends. **(D)** The 25 keywords with the highest burst strength ordered chronologically. **(E)** The 25 keywords with the highest burst strength ordered by strength.

The evolutionary path of the field follows a clear trajectory from foundational concepts toward specific applications, marked by continuous differentiation and integration. A comprehensive analysis of the temporal keyword Sankey diagram, thematic trend graphs, and burst words (Figure 15) traces the field’s historical development. Early research (circa 1976–2000) primarily revolved around relatively macroscopic biological phenomena such as “stress,” “immune response,” and “integrin” (Holmvall et al., 1995; Pedersen et al., 1999; Jones et al., 2000). In the 21st century, the research focus significantly shifted and deepened, forming core research branches represented by “mechanotransduction,” “extracellular matrix,” and “stiffness,” which became closely linked with key processes in cancer biology such as “metastasis” and “migration.” Since 2015, the research front has rapidly pivoted toward directions with greater clinical value: the prominence of emerging fields such as “mechanobiology” and “cancer immunotherapy” is rapidly increasing, while research into molecular mechanisms such as “YAP-signaling proteins” and the “Piezo1” ion channel has become a new growth point (Pontes and Mendes, 2023; Qu and Zhang, 2025). Future research is expected to focus more intently on the roles of these molecular mechanisms in specific pathological contexts, such as “solid stress” and the mechanical regulatory networks in specific diseases such as “ovarian cancer” and “colorectal cancer.” This outlook signals the field’s progression toward deeper mechanistic exploration and clinical translational applications.

## 4 Discussion

### 4.1 Data analysis findings

This study provides a panoramic depiction of the knowledge structure, evolutionary dynamics, and global collaboration landscape of the interdisciplinary field at the intersection of mechanobiology and cancer. As a multidisciplinary domain, tumor mechanobiology has shown significant growth in recent years and has considerable value in understanding and treating cancer (Linke et al., 2024). Although traditional oncology research has long focused on molecular and genetic mechanisms, the role of mechanical forces in regulating the tumor microenvironment has been comparatively overlooked. Recently, however, with the development of detection methods such as single-cell force spectroscopy and atomic force microscopy, the critical role of mechanical forces in tumor cell proliferation, invasion, metastasis, and drug resistance has been increasingly recognized, establishing tumor mechanobiology as a key frontier in cancer research (Li, 2024).

Despite the growing interest in this field, previous bibliometric analyses have primarily mapped the broader and generalized landscape of mechanobiology (Dong D.-L. et al., 2024). These existing studies often rely on single databases (such as PubMed) over relatively short time spans (e.g., 2010–2024), yielding an overview of general biomechanics and tissue engineering. Consequently, they fail to capture the specific biological nuances, complex molecular mechanisms, and emerging clinical translations that are unique to oncology.

To address this critical knowledge gap, our study is exclusively dedicated to ‘cancer mechanobiology’ and significantly extends the temporal scope to half a century (1976–2025). Methodologically, unlike previous studies constrained by cross-database heterogeneity, we engineered a novel custom multi-stage Python-based data standardization workflow to rigorously integrate 1,947 high-quality publications from both the WoS and Scopus databases. This rigorous data harmonization ensures an unprecedented level of data fidelity, enabling our multi-modal network analysis to construct the first high-fidelity comprehensive knowledge map specifically focused on the physical tumor microenvironment and its immunotherapeutic implications.

#### 4.1.1 Ensuring the validity of cross-database analysis

The validity of this discussion is predicated on the reliability of the underlying data analysis. To address the pervasive challenge of data heterogeneity between the Scopus and WoS databases, this study constructed and implemented an innovative, multi-stage, semi-automated data cleaning and standardization workflow. This process primarily involved multiple rounds of manual review and matching to achieve a high degree of format normalization for the C1, C3, RP, CR, J9, and PY fields. Furthermore, a unified thesaurus and stop-word list were constructed for VOSviewer and CiteSpace, with this information also being applied within Bibliometrix. This rigorous methodology substantially enhances the accuracy and credibility of the analysis presented herein.

#### 4.1.2 From macroscopic phenomena to microscopic mechanisms and clinical translation in tumor mechanobiology

Keyword and cited reference analyses clearly show that early research (circa 1976–2000) focused on relatively macroscopic biological concepts such as “stress” and “immune response” (Pedersen et al., 1999; Jones et al., 2000). Subsequently, the intellectual core of the field gradually converged and deepened. The keyword co-occurrence network and high-frequency word analysis jointly reveal that “mechanotransduction,” “extracellular matrix,” and “cancer” form the undisputed “iron triangle” research framework of the domain. The establishment of this framework aligns perfectly with the timeline of foundational works identified in the highly-cited reference analysis, such as those by Paszek et al. (2005) and Levental et al. (2009). These pioneering studies revealed the regulatory role of matrix stiffness in tumor malignancy, marking the formation of the field’s core research paradigm. As research progressed, keyword burst analysis captured a further deepening from macroscopic physical properties toward the exploration of key molecular mechanisms. Molecular keywords such as “YAP-signaling proteins” and the “Piezo1” ion channel rapidly became research hotspots after 2015 (Dombroski et al., 2021; Mukherjee et al., 2025), a trend that corroborates the emergence of scholars like Dupont S. as the core highly co-cited authors (Dupont and Wickström, 2022).

Currently, the evolutionary trajectory of the field is clearly pointing toward clinical translation. Keyword burst analysis shows that terms with explicit clinical applications, such as “cancer immunotherapy” and “solid stress,” have become the highest-intensity burst words in recent years (Kalli et al., 2023; Yuan et al., 2023). This trend is cross-validated by the results of the dual-map overlay analysis: the knowledge flow moves robustly from the “molecular biology/immunology” cluster on the left, representing foundational disciplines, to the “molecular biology/genetics” cluster on the right, representing the applied frontier. This evolution from basic to applied science is reflected not only in research themes but also in publication venues, as research findings are increasingly published not just in traditional biology journals but also in emerging interdisciplinary technology journals such as *Nature Biomedical Engineering*. The cross-pollination of this field with molecular biology, materials science, and immunology also indicates that future research is likely to transcend single-disciplinary boundaries.

#### 4.1.3 Asymmetry in national output and collaboration networks

At the macro level, the global research output in this field presents a highly concentrated bipolar structure, co-dominated by the United States and China. However, a cross-comparison of publication volume with the collaboration network structure reveals a significant asymmetry. The United States not only leads in the number of publications and citations but its institutions (such as the University of California System) also serve as central hubs in the international collaboration network, possessing the highest degree and betweenness centrality.

In stark contrast, although China is firmly second in academic output, its role as a knowledge broker within the global network remains underdeveloped relative to its output, thereby exhibiting a characteristic of “high output, weak connectivity.” Its network centrality, particularly its betweenness centrality as a collaborative bridge, is relatively low, indicating that its research is more concentrated within regional clusters or in collaborations with a few core nodes. The high burst strength of Chinese institutions (e.g., Fudan University) indicates immense developmental potential, but the key challenge for the future will be to effectively translate this vast research volume into network influence and global leadership. This landscape indicates that international collaboration in the field is still predominantly led by traditional scientific powers in Europe and North America, and a more balanced and multi-centric global collaboration network has yet to fully emerge.

### 4.2 Tumor mechanics in therapy

#### 4.2.1 Mechanisms of the mechanical microenvironment and potential for cancer therapy

The TME is intimately involved in cancer initiation and progression (de Visser and Joyce, 2023). Rather than a static structure, the TME is a dynamic system that co-evolves with the tumor. It is composed of distinct yet interacting microenvironments—physical (e.g., oxygen and pH), mechanical (e.g., ECM and vasculature), metabolic (e.g., glucose and lipids), inflammatory, and immune—that profoundly influence tumorigenesis and tumor development (Wang Y. et al., 2025). A complex network of mechanisms involving hypoxia, chronic inflammation, and immunosuppression has been identified, making these processes critical targets for cancer therapy. For instance, the metabolic state of myeloid-derived suppressor cells (MDSCs) within the TME determines their immunosuppressive function, indicating that the modulation of MDSC metabolism is a viable strategy to enhance anti-tumor immunity (Wang H. et al., 2025). The accumulation of reactive oxygen species (ROS), by-products of aerobic metabolism, is a hallmark feature of many cancers. By modulating cell proliferation, metastasis, and angiogenesis, ROS are critical to tumor progression; thus, therapeutic strategies targeting ROS in cancer cells and the TME represent a promising future direction (Shen et al., 2025). Tumor-associated macrophages (TAMs) constitute the principal inflammatory cell population in the TME. Cancer-associated fibroblasts (CAFs) can recruit monocytes and induce their differentiation into M2-like TAMs via various cytokines, which in turn can augment the efficacy of immunosuppressive therapies (Jia et al., 2025).

As a key component of the TME, the ECM performs multiple fundamental functions, which range from providing mechanical support and shaping the microenvironment to modulating metabolism, signal transduction, and immune responses. Consequently, the study of the ECM is a major focus of tumor mechanobiology. Within the tumor, the ECM often undergoes stiffening, which activates cellular mechanotransduction pathways (Sleeboom et al., 2024) and fosters hypoxic and metabolically challenging conditions. The ECM is also instrumental in regulating cancer invasiveness by enhancing tumor heterogeneity and cancer cell stemness, which are critical drivers of metastasis and therapeutic resistance (Finger et al., 2025). For example, a study on bladder cancer found that the E26 transformation-specific (ETS) transcription factor GA-binding protein transcription factor subunit alpha (GABPA) promotes collagen cross-linking and ECM deposition, thereby remodeling the matrix to facilitate tumor progression (Dai et al., 2025). Such findings, along with a growing body of similar evidence, underscore the potential of targeting ECM regulation as a therapeutic strategy.

Although the ECM presents considerable therapeutic potential, some researchers argue that its degradation, while disrupting the tumor-supporting scaffold, could conversely create a more permissive environment for cancer cell migration and invasion (Zhang X. et al., 2025). Current research predominantly focuses on single mechanical parameters, which fail to replicate the multi-scale mechanical coupling of the ECM *in vitro*. Therefore, future biomaterial design must be predicated on a deeper understanding of the mechanical heterogeneity that is inherent to different tumor ECMs and should integrate multiple mechanical parameters to more accurately recapitulate the complex *in vivo* mechanical microenvironment (Huang et al., 2025).

#### 4.2.2 The mechanical microenvironment and cancer detection

The study of the mechanical microenvironment also has applications in cancer detection. Because solid tumors exhibit abnormal biomechanical properties, these characteristics can be measured to assess the invasive or metastatic potential of cancer cells and evaluate the efficacy of therapeutic agents.

Currently, established and refined methods for examining tissue and cell mechanics can be broadly categorized into five major types. Atomic force microscopy (AFM) uses a nanoscale probe to scan a sample’s surface, enabling the measurement of mechanical properties such as elastic modulus and viscoelasticity, while simultaneously providing high-resolution imaging. This technique is suited for the micromechanical analysis of single cells, biofilms, and soft materials, such as in studying stiffness changes in cancer cells or the interaction forces between protein molecules. Its advantages lie in its nanoscale precision and non-destructive nature, but it has limitations in characterizing complex three-dimensional structures (Miranda et al., 2021). Ultrasound shear wave elastography (SWE) utilizes ultrasonic waves to induce shear waves within tissue. Tissue stiffness can be inferred by measuring the propagation speed of these waves. SWE is applicable for the clinical diagnosis of conditions such as liver fibrosis and muscle diseases. The technique allows rapid, real-time, and non-invasive acquisition of large-area elasticity maps *in vivo*, but its utility is constrained by the penetration depth of sound waves and tissue heterogeneity (Suffredini et al., 2024). Magnetic resonance elastography (MRE) integrates magnetic resonance imaging with mechanical wave excitation. By analyzing tissue vibration patterns, it reconstructs three-dimensional elastography maps and is commonly used to evaluate mechanical abnormalities in organs such as the liver and breast. The primary advantages of MRE are its high spatial resolution and its ability to penetrate deep tissues; however, it is limited by high equipment costs and operational complexity (Meyer et al., 2024). Microfluidic technologies manipulate small volumes of fluid within microchannels to simulate cellular microenvironments and analyze cellular mechanical responses. Applications include single-cell sorting, drug screening, and the construction of organ-on-a-chip models. This approach allows precise control over parameters such as fluid shear stress and pressure gradients and is suitable for high-throughput analysis, although the biomimicry of complex tissues requires further improvement. Additionally, there are other emerging techniques, but they have minimal clinical application and remain primarily in the laboratory research phase (Hong et al., 2025).

In the future, multidisciplinary collaboration is expected to promote the development of combination therapies and refine current treatment strategies. The integration of tumor mechanobiology is anticipated to ultimately yield more effective and personalized therapeutic approaches, addressing the complexity of tumors and improving patient outcomes (Angeli et al., 2025).

#### 4.2.3 Synergistic mechano-immunotherapy

Tumor cells utilize various strategies to diminish the efficacy of immunotherapy, including increasing the stiffness of the ECM, which contributes to poor clinical outcomes in some patients. Increased ECM stiffness and a dense collagen network can impede the infiltration and migration of immune cells, thereby impairing cytotoxic interactions between immune cells and cancer cells (Peranzoni et al., 2013). A growing body of clinical evidence now supports mechano-targeting as a strategy to enhance therapeutic efficacy. For example, the efficacy of pirfenidone in regulating lung cancer by inhibiting transforming growth factor-beta (TGF-β) has been validated in clinical trials (Muppala et al., 2017). Similarly, the monoclonal antibody C1-3, which specifically targets a transmembrane protein expressed by hepatic myofibroblasts, induces myofibroblast apoptosis and significantly reduces the severity of fibrosis when used in combination with gliotoxin (Navarro et al., 2024). Furthermore, animal studies have identified a critical interplay between extrinsic matrix stiffness and intrinsic sphingolipid synthesis regulated by insulin-like growth factor 2 mRNA-binding protein 2 (IGF2BP2), presenting a promising target for immunotherapy strategies in pancreatic ductal adenocarcinoma (Tang et al., 2025).

In summary, current synergistic mechano-immunotherapies are centered on the modulation of mechanical forces. By linking these mechanical inputs to multiple immune cell targets and combining them with existing immunotherapies, the goal is to overcome TME immunosuppression and amplify the anti-tumor immune response. Current research in this area can be broadly categorized into three synergistic approaches (Ren et al., 2025).

The first approach involves the synergistic modulation of mechanical forces and multiple immune-cell targets. A single mechanical force within the TME, such as matrix stiffness, can concurrently affect the function of T cells, natural killer (NK) cells, and macrophages. Therefore, targeting the origin of this mechanical force can achieve synchronous, multi-cellular regulation. This strategy primarily encompasses ECM softening and vascular normalization. Softening the ECM using inhibitors of lysyl oxidase or matrix metalloproteinase (MMP) modulators can alleviate T-cell exhaustion and migratory inhibition while also breaking down the ‘mechanical barrier’ for NK cells, thus promoting their infiltration. This approach can also suppress the M2 polarization of macrophages and promote their conversion to an M1 phenotype (Chen et al., 2020). This foundational strategy can serve as a prerequisite for other regulatory interventions. Vascular normalization, achieved through anti-angiogenic drugs such as anti-vascular endothelial growth factor (VEGF) agents, improves tumor vascular morphology, reduces solid stress, and optimizes fluid shear stress. These changes can alleviate hypoxia, reduce T-cell and NK-cell inactivation, enhance NK-cell extravasation into the tumor parenchyma, and decrease neutrophil adhesion to vessels, thereby preventing the formation of an immune barrier (Dallavalasa et al., 2021).

The second approach targets mechanosensitive molecules, such as Piezo1, which act as critical regulatory nodes across different cell types. Targeting these molecules can complement existing immunotherapies such as chimeric antigen receptor T-cell (CAR-T) therapy and immune checkpoint inhibitors (ICI), thereby significantly enhancing therapeutic outcomes. For instance, in a synergistic strategy combining Piezo1 targeting with ICI, Piezo1 activation in T cells promotes programmed cell death protein 1 (PD-1) expression via the Ca^2+^/nuclear factor of activated T-cell (NFAT) pathway, while in macrophages, it drives polarization toward the pro-tumorigenic M2 phenotype. Inhibition of Piezo1 not only reduces the PD-1 levels on T cells but also suppresses the pro-tumor function of TAMs. When combined with PD-1/programmed death-ligand 1 (PD-L1) inhibitors, this approach can achieve a dual reversal of TME immunosuppression, thereby enhancing the tumor-killing efficiency of T cells. In a separate synergy, Piezo1 targeting can be combined with NK-cell therapy. Piezo1 directly regulates intracellular calcium flux and the activation process in NK cells. By optimizing Piezo1 activity, the functional stability of NK cells can be enhanced within the abnormal fluid-shear-stress environment of the TME. Combining this with adoptive NK-cell therapy can significantly improve the survival and cytotoxicity of NK cells in solid tumors (Xing et al., 2024).

The third approach utilizes physical stimulation technologies, such as ultrasound and photothermal therapy, which offer remote and spatiotemporally precise modulation of TME mechanical forces or immune cell functions. Combining these techniques with therapies such as CAR-T can effectively reduce off-target toxicity. In a strategy combining physical stimulation with CAR-T, ultrasound or photothermal techniques can be used to locally modulate ECM stiffness or directly activate photo-controllable CAR-T cells. This allows precise, localized proliferation and activation of CAR-T cells at the tumor site, avoiding the cytokine storms associated with systemic overactivation in normal tissues while also enhancing the ability of CAR-T cells to penetrate solid tumors (Luo et al., 2022). Another synergy involves the regulation of neutrophil extracellular trap (NET) formation (NETosis). Photothermal techniques can be used to precisely control local NETosis, avoiding the inflammatory risks of excessive systemic NET production while leveraging the ability of NETs to trap tumor cells, thereby working in concert with NK-cell or T-cell therapies to clear tumors (Khanmohammadi et al., 2024).

### 4.3 Strengths and limitations of this study

This study possesses several notable strengths that distinguish it from previous reviews. Methodologically, it successfully overcomes the pervasive challenge of cross-database heterogeneity by systematically integrating literature from both the WoS Core Collection and Scopus. Rather than relying on a single database, which often introduces coverage bias, we developed a novel, customized multi-stage Python parsing engine. This rigorous data standardization workflow achieved a 99.4% success rate in reference conversion and effectively harmonized critical metadata fields across platforms. Consequently, the resulting dataset offers exceptional fidelity and breadth. Furthermore, by synergistically utilizing multiple advanced analytical tools (CiteSpace, VOSviewer, and Bibliometrix), this study provides a multi-dimensional perspective—ranging from static topological networks to dynamic temporal alluvial flows—thereby capturing the complex evolutionary trajectory of cancer mechanobiology with high resolution.

However, certain limitations must be explicitly acknowledged. First, despite leveraging two major comprehensive databases, the exclusion of other specialized repositories (such as PubMed, Embase, or preprint servers such as bioRxiv) and an inherent bias toward English-language publications may mean that some relevant regional or highly specialized literature was omitted. Second, bibliometric analyses naturally suffer from a citation time-lag phenomenon. Groundbreaking studies published very recently may not yet have accumulated sufficient citation metrics to emerge as central nodes in co-citation networks, potentially leading to an underrepresentation of the most recent frontier dynamics. Finally, quantitative co-citation and keyword analyses cannot definitively distinguish the specific semantic context of a citation. Future research could integrate advanced natural language processing (NLP) techniques for deep semantic analysis of full-text citation contexts, thereby providing a more nuanced evaluation of knowledge flow and scientific consensus in tumor mechanomedicine.

While this study offers a comprehensive bibliometric mapping of cancer mechanobiology, several inherent limitations warrant acknowledgment. First, despite integrating both Web of Science and Scopus, these databases show a well-documented bias toward English-language publications, potentially underrepresenting contributions indexed in regional platforms such as China National Knowledge Infrastructure (CNKI), Embase, or J-STAGE (Mongeon and Paul-Hus, 2016; Aksnes and Sivertsen, 2019). Future work should expand the corpus to include multilingual and platform-diverse sources. Second, although our multi-stage standardization pipeline achieved a 99.4% reference conversion success rate, residual inconsistencies in author name disambiguation and institutional affiliation normalization cannot be entirely eliminated, which may introduce minor biases into co-authorship and citation analyses (Franceschini et al., 2016). Third, and most fundamentally, bibliometric analysis is inherently correlational rather than causal. Our findings identify associations among publication trends, collaboration patterns, and keyword co-occurrence but cannot establish causality or distinguish genuine conceptual influence from citation bias. The structural maps we constructed reflect the field’s momentum at a given point in time rather than explaining the mechanisms underlying its trajectory. Future investigations should, therefore, complement this macro-level perspective with fine-grained mechanistic inquiry—including qualitative content analyses of high-burst literature clusters, multi-scale temporal analyses at shorter intervals, and mixed-methods approaches that triangulate bibliometric evidence with clinical trial databases and patent records.

The collaboration asymmetry identified in our analysis—wherein China ranks second globally in publication volume yet maintains relatively low betweenness centrality—reflects the structural features of the broader Chinese research ecosystem. National Science Foundation (NSF) data indicate that China’s international collaboration rate stood at only 19% in 2022, far below the United States (40%), the United Kingdom (67%), and Germany (56%), with domestically-dominated publishing clusters further limiting network integration (Marginson and Xu, 2023). Contributing factors include volume-oriented incentive structures, concentrated institutional funding, geopolitical headwinds, and linguistic barriers—patterns similarly observed in other rapidly rising scientific powers such as India and South Korea (Bornmann et al., 2014).

Narrowing this structural gap will require concerted efforts at multiple levels. Bibliometric evidence demonstrates that internationally mobile Chinese scholars serve as critical bridging nodes upon return (Wang C. et al., 2024), indicating that pursuing co-led international projects and joint corresponding authorships in high-impact interdisciplinary journals would meaningfully enhance network centrality. Institutionally, establishing joint laboratories with high-centrality nodes identified in our analysis—particularly within the University of California System and the University of Pennsylvania—and adopting multi-principal investigator (PI) grant mechanisms modeled on NSF–National Natural Science Foundation of China (NSFC) bilateral programs would catalyze the sustained collaborations necessary for genuine global leadership. Given the field’s imminent clinical translation, this strategic transition from high-volume domestic output to internationally agenda-setting science is both feasible and urgent.

In conclusion, novel combination treatment protocols based on the mechano-regulation of immunotherapy are poised to become a major research focus. These synergistic mechano-immune therapies will likely integrate our understanding of the tumor microenvironment and mechanobiology with advanced platforms such as organoids and organ-on-a-chip models (Park et al., 2025).

## 5 Frontiers and prospects

The theoretical foundations of tumor mechanobiology can be traced to the early 20th century, when D’Arcy Thompson pioneered the interdisciplinary study of morphogenesis. In his book *On Growth and Form*, he proposed that the principles of physics and mathematics could explain the formation of biological structures, thereby laying the theoretical groundwork for mechanobiology. However, due to the limitations of the era, the field experienced little significant progress for several decades.

With subsequent technological advancement, the advent of tools such as AFM and optical tweezers in 1986 provided the essential experimental support for mechanobiology research (Binnig et al., 1986). The application of traction force microscopy to cells on various substrates further advanced the study of cell mechanics (Oliver et al., 1995). Supported by this theoretical and technological maturation, the concept of tumor mechanobiology began to germinate. Bissell et al. (1982) proposed the “dynamic reciprocity” hypothesis (Bissell et al., 1982), demonstrating that the ECM and its receptors determine the phenotype of mammary epithelial cells and confirming the existence of a “dynamic dialogue” between the ECM and the cell nucleus (Weaver et al., 1997).

Paszek and Weaver (2004) explicitly linked mechanics with oncology, first proposing the concept of tumor mechanics. In a subsequent publication, they introduced the concept that matrix stiffness dictates cell fate, linking it to cytoskeletal tension and establishing an integrated mechano-regulatory circuit involving extracellular signal-regulated kinase (ERK) and Rho, which set the tone for future research directions in tumor mechanobiology (Paszek et al., 2005).

The ECM, as a critical component, has received significant attention within the field. The ECM of varying elasticity has been shown to induce the differentiation of stem cells into different lineages (Engler et al., 2006), and collagen cross-linking has been demonstrated to enhance ECM stiffness and promote epithelial cell invasion (Levental et al., 2009). For some time, however, the precise mechanisms by which mechanical signals are transduced to affect cells remained elusive. Research into the YAP/TAZ pathway, dating back to 2011, provided a breakthrough when Dupont et al. (2011) first established the Hippo-independent mechanosensitivity of YAP and TAZ. This discovery opened a new research area focused on understanding the influence of the TME’s mechanical properties on cancer development, with YAP/TAZ acting as the key mechanotransducers (Dupont et al., 2011).

With increasingly significant findings and impact, tumor mechanobiology has gained recognition as a new interdisciplinary field (Ladoux and Mège, 2017; Chaudhuri et al., 2018; Roy Choudhury et al., 2019). As a new frontier in cancer research (Rao et al., 2021; Yu et al., 2022), tumor mechanobiology, supported by a continuously strengthening foundation of basic research, is progressively being translated into clinical medicine (Dong Y. et al., 2024).

The rise of tumor mechanomedicine reflects a trend toward multidisciplinary integration. By combining advanced technologies such as single-cell sequencing, high-resolution imaging, and artificial intelligence algorithms, the mechanical properties and signaling networks of the TME will be analyzed with unprecedented precision. The influence of the YAP/TAZ pathway on diverse tumor types and its crosstalk with classical oncogenic pathways will likely be further elucidated (Pontes and Mendes, 2023). Additional mechanosensitive molecular networks will be systematically described, offering new therapeutic avenues for refractory tumors and for overcoming drug resistance (Kalli et al., 2023; Qu and Zhang, 2025).

## Data Availability

The original contributions presented in the study are included in the article/supplementary material; further inquiries can be directed to the corresponding author.
